# Cylindrical Metalens for Generation and Focusing of Free-Electron Radiation

**DOI:** 10.1021/acs.nanolett.1c04556

**Published:** 2022-07-06

**Authors:** Aviv Karnieli, Dolev Roitman, Matthias Liebtrau, Shai Tsesses, Nika Van Nielen, Ido Kaminer, Ady Arie, Albert Polman

**Affiliations:** † Raymond and Beverly Sackler School of Physics and Astronomy, Tel Aviv University, Tel Aviv 69978, Israel; ‡ Center for Nanophotonics, NWO-Institute AMOLF, Science Park 104, 1098 XG Amsterdam, The Netherlands; § Andrew and Erna Viterbi Department of Electrical Engineering, Technion−Israel Institute of Technology, Haifa 32000, Israel; ∥ School of Electrical Engineering, Fleischman Faculty of Engineering, Tel Aviv University, Tel Aviv 69978, Israel

**Keywords:** nanophotonics, metasurfaces, metalens, wavefront shaping, free electrons, cathodoluminescence, Smith−Purcell radiation

## Abstract

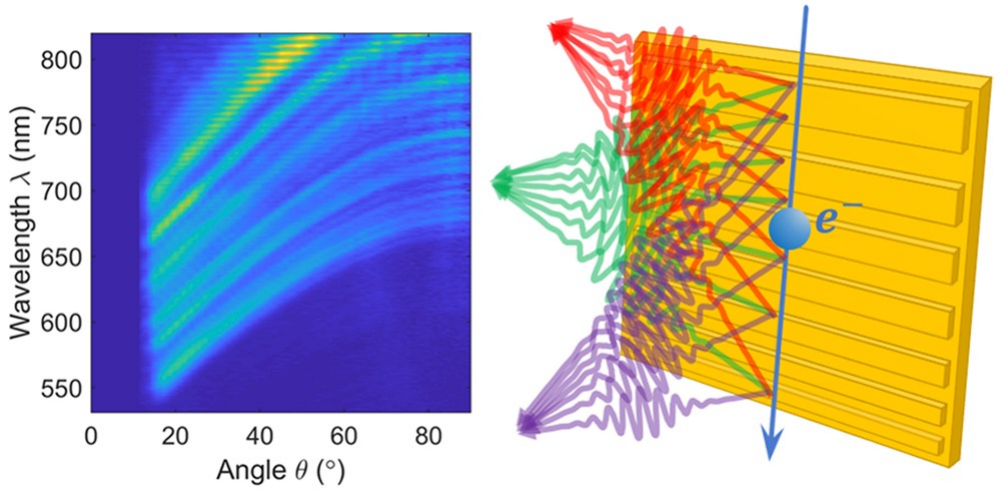

Metasurfaces constitute a powerful approach to generate and control light by engineering optical material properties at the subwavelength scale. Recently, this concept was applied to manipulate free-electron radiation phenomena, rendering versatile light sources with unique functionalities. In this Letter, we experimentally demonstrate spectral and angular control over coherent light emission by metasurfaces that interact with free-electrons under grazing incidence. Specifically, we study metalenses based on chirped metagratings that simultaneously emit and shape Smith–Purcell radiation in the visible and near-infrared spectral regime. In good agreement with theory, we observe the far-field signatures of strongly convergent and divergent cylindrical radiation wavefronts using *in situ* hyperspectral angle-resolved light detection in a scanning electron microscope. Furthermore, we theoretically explore simultaneous control over the polarization and wavefront of Smith–Purcell radiation via a split-ring-resonator metasurface, enabling tunable operation by spatially selective mode excitation at nanometer resolution. Our work highlights the potential of merging metasurfaces with free-electron excitations for versatile and highly tunable radiation sources in wide-ranging spectral regimes.

Metasurfaces provide unprecedented control over light down to the sub-micrometer scale,^1−5^ facilitating planar optical components for imaging^6,7^ and focusing,^8−10^ polarization control,^11^ wavefront shaping,^12,13^ analog image processing,^14−16^ and holography.^17−19^ In addition, metasurfaces can be used to manipulate the emission of light itself, enabling processes such as nonlinear frequency conversion,^20−24^ spin–orbit-coupled lasing,^25^ and tailored quantum-light generation.^26,27^ The key to all these functionalities lies in a subwavelength-scale surface pattern, hosting localized excitations that collectively define the desired scattering response. Typically, this design is tailored to far-field illumination schemes.^8−10^ However, metasurfaces also render control over near-field excitation mechanisms^26,27^ with a plethora of possibilities yet to be explored.

One such approach is to embed metasurfaces with free-electron radiation phenomena. Free electrons can directly couple to optical excitations in nanophotonic structures, providing a powerful tool to generate radiation with unique spectral, spatial and temporal properties.^28−34^ Remarkably, this interaction typically occurs on femto- or attosecond time scales,^35−42^ yielding access to spectral regimes in which conventional radiation sources are usually unavailable or inefficient, such as the terahertz (THz),^43^ ultraviolet (UV),^44^ or X-ray^45^ ranges. Moreover, the electron wave function can be confined to length scales many orders of magnitude below the emission wavelength, facilitating free-electron-driven metasurfaces with a multitude of functionalities that are tunable by nanometric structural parameters, the electron excitation path, as well as the electron energy.

Previous work has demonstrated the emission of structured light by metasurfaces that interact with a free-electron beam under normal incidence, including effects such as lensing, vortex beam generation, and polarization control.^33,46−49^ However, in this configuration the electron–light coupling is typically weak due to a short electron–sample interaction range as compared to the excitation wavelength. In contrast, grazing excitation schemes permit each electron to coherently drive several consecutive emitters in a metasurface along the electron trajectory, enabling electron-light phase matching—i.e., a match between the electron velocity and phase velocity of the induced optical field.^31,50^ The benefits of such phase-matched interactions are 3-fold. First, the incident electron energy provides an effective and easily accessible tuning knob to generate radiation over an ultrawide spectral range. Second, the intensity of the emitted light can be significantly enhanced by increasing the number of unit cells that are interacting with the electron, also permitting emission into a well-defined spectral range. Finally, different functionalities can be encoded into a single metasurface by varying structural parameters such as the periodicity, duty cycle (fill factor), and unit cell morphology.

A well-known example for phase-matched interactions between free electrons and light is the Smith–Purcell (SP) effect.^29,51,52^ The SP effect describes broad-band emission of radiation into discrete diffraction orders by a free electron that grazes a periodic grating. Interestingly, studies in recent years have shown that periodic nanostructures can be engineered to control the spectral^53^ and polarization^54,55^ properties of SP radiation. However, the greater challenge of sculpting the *spatial* and *angular* distribution of the emission has only been explored in theory thus far^53,56−59^ or in analogous experiments.^60^ In particular, the challenge of *focusing* SP radiation has been pursued theoretically by many researchers,^53,56,57,61^ motivated by the demand for effective lenses and other optical components in technically challenging spectral regimes, such as the extreme UV or soft X-rays.

In this work, we provide the first experimental demonstration of a metasurface lens (metalens) for phase-matched free-electron light emission. Specifically, our structures are designed to transform the conventional SP plane-wave emission into cylindrical wavefronts with a convex or concave curvature for light in the visible (VIS) and near-infrared (NIR) spectral regimes. The radiation profiles of converging and diverging metalenses are characterized by *in situ* hyperspectral angle-resolved light detection^62^ in a scanning electron microscope, enabling a distinct analysis of the two lens types through their far-field signatures. Remarkably, we achieve a coherent electron–sample interaction over more than 100 metagrating periods, resulting in the emission of light within effective numerical apertures (NAs) of 0.48 ± 0.05 and 0.45 ± 0.05 for the converging and diverging metalenses, respectively.

To further expand the metalens concept, we present numerical simulations of a split-ring-resonator (SRR) metasurface, yielding simultaneous *focusing* and *polarization control* by shifting the lateral position of the electron beam with respect to the SRR meta-atoms. Our work demonstrates the wide range of possibilities that are enabled by merging metasurfaces with phase-matched free-electron radiation mechanisms. Complementary to traditional illumination schemes, this new approach holds a unique potential for tunable emission of radiation with arbitrarily tailored spatial, spectral, and polarization properties in challenging and technologically relevant wavelength regimes.

To reproduce the conceptual basis of our SP metalenses as proposed in previous work,^53,56,57,61^ let us first recall the theory of the conventional SP effect.^29^ The far-field radiation emitted by an electron of normalized velocity β = *v*/*c* that passes near a grating with constant period Λ fulfills the characteristic dispersion relation^29^
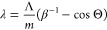
1where λ is the emission wavelength, *m* is the diffraction order, and Θ is the elevation angle with respect to the direction of propagation of the electron, hereinafter referred to as the *z* direction. This dispersion relation is the direct result of energy and momentum conservation,^51^ assuming that light is emitted as a plane wave. Notably, eq 1 represents a phase-matching condition between the electron and the grating near field, with the grating periodicity providing the necessary excess momentum to mediate an energy transfer from the electron to a photon scattered into free space. A more detailed discussion of this condition can be found in Section 2 of the Supporting Information.

In general, for light of nominal wavelength λ_0_ to be emitted into an arbitrary phase front ϕ(*z*),^53,56^ the emission angle Θ defined by eq 1 varies along the electron trajectory as cos Θ (*z*) = (λ_0_/2π) dϕ(*z*)/d*z*. Such a variation can be induced by a structure that scatters waves with a spatially dependent excess momentum, as described by a generalized formulation of Snell’s law for metasurfaces.^3,4^ From eq 1 the required modulation Λ(*z*) of the grating periodicity then follows as
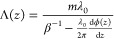
2

Notably, the conventional SP relation is again recovered assuming a linear phase front ϕ(*z*) = 2π*z* cos Θ/λ_0_ (i.e., a plane wave propagating at an angle Θ with respect to the electron direction of propagation).

The SP metalenses^53,56,57,61^ studied in this work are intended to emit light into a cylindrical wavefront with a phase profile , where ±*f* is the nominal focal distance of the lens with respect to the grating plane. The emission is then focused or defocused in the *x*–*z* plane perpendicular to the grating plane while retaining azimuthal divergence along the *y* direction. Accordingly, eq 2 yields a chirped periodicity of the form
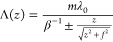
3which, in practice, we implement by sampling Λ(*z*) over a range of discrete values *z*_*n*_, with the *n*th periodicity evaluated at *z*_*n*_ = *z*_*n*–1_ + Λ(*z*_*n*–1_). Furthermore, we choose to maintain a constant 50% duty cycle (i.e., fill factor), such that the width of each grating element scales in proportion to Λ(*z*), as depicted in Figure 1.

**Figure 1:**
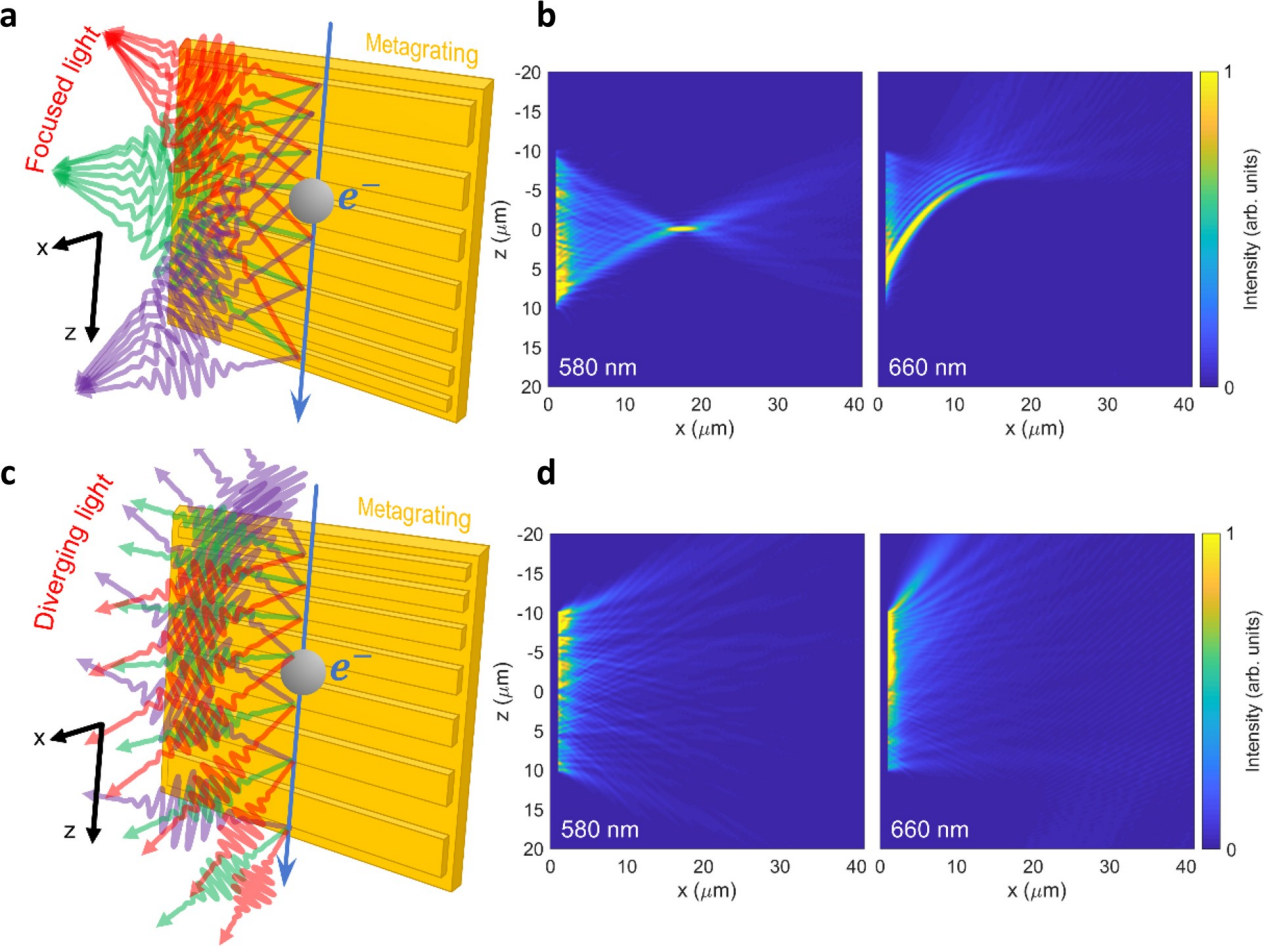
Focused Smith–Purcell emission by a free-electron-driven metalens: concept and simulation. (a) Free electrons move along the *z* direction and pass in close proximity to a metagrating with a chirped period. Light is emitted into a converging wavefront, with different wavelengths converging at different positions in the *x*–*z* plane. The nominal wavelength λ_0_ (green rays; see eq 3) is focused 17 μm away from the grating plane and opposite its center. Shorter (longer) wavelengths, marked by purple (red) rays, are focused closer to the grating and below (above) its center. (b) Normalized full-wave three-dimensional FDTD simulation of the near-field emission pattern using the structure in (a), at wavelengths of 580 nm (left) and 660 nm (right), as induced by a 30 keV electron beam incident a few nanometers away from the structure. The nominal metagrating wavelength (λ_0_ = 580 nm) shows perfect focusing at an NA value of 0.5, whereas other wavelengths (λ_0_ = 660 nm shown for reference) are focused more weakly via caustics. The model grating is 20 μm in length and varies in period according to eq 3, from 228 to 163 nm. (c) The same as in (a), yet with an inverted chirp, resulting in a divergence of the emitted SP radiation (as opposed to focusing). The direction of divergence for shorter and longer wavelengths is inverted as well. (d) Normalized full-wave FDTD simulation of the diverging wavefront emitted by the structure in (c), using the same parameters as in (b). The emitted light diffracts as if its focal plane were located 17 μm behind the grating surface. The design of the metalenses emitting cylindrical radiation wave fronts , with *f* and λ_0_ as specified above, is based on eq 3.

Figure 1a schematically illustrates the operation of a converging metalens. For the nominal design wavelength λ_0_, the lens emits light into a focal spot at *z* = 0 and *x* = *f*, while for any other wavelength, the focus moves to a different position within the *x*–*z* plane. Intuitively, this phenomenon can be understood by considering the *local* emission of SP radiation from finite subsections of the grating structure at a given wavelength λ. In this picture, the rays emitted from *z* < 0 toward the nominal focus at (0, *f*) form an acute angle with respect to the electron trajectory, those emitted near *z* = 0 form a right angle, and those emitted from *z* > 0 form an obtuse angle. However, waves that are scattered under a given angle can only exist if the local excess momentum provided by the grating permits phase matching with the electron. For the rays intersecting at the desired focal point, only light of the nominal wavelength λ_0_ interferes constructively, while light emitted at smaller and larger wavelengths is focused below or above the optical axis, respectively. For further information on the metalens operation, the reader is referred to Sections 1 and 2 of the Supporting Information.

The converging or diverging character of the SP metalens, and hence the curvature of the emitted wavefronts, is directly related to the orientation of the grating chirp as determined by the sign of ϕ(*z*). Thus, for the same values |*f*| and λ_0_ (as specified in the caption of Figure 1), the diverging or converging metalenses simply differ by an inverted chirp. The NA of the SP metalens is ideally given by , where *L* is the total physical length of the grating. Yet, we note that in an experiment the coherent interaction length of an electron with the structure *L*_eff_ can be reduced due to a nonideal beam–sample alignment or a finite beam divergence, practically limiting the effective NA. Due to the grating chirp, a finite interaction length further affects the spectral emission pattern, yielding a distinct difference in the far-field profile of the converging and diverging metalenses as discussed below and elaborated further in Section 3 of the Supporting Information.

To support the above theoretical discussion, complementary numerical simulations were performed using a full-wave finite-difference in the time-domain (FDTD) solver (Lumerical, Ansys Cananda Inc.). Figure 1b,d shows the simulated near-field patterns of a converging and a diverging metalens with focal lengths of *f* = ±17 μm for a nominal emission wavelength of λ_0_ = 580 nm and 30 keV electron-beam excitation (β = 0.328), assuming an interaction length of *L* = 20 μm. The two simulations show the induced near-field patterns at wavelengths of 580 nm and 660 nm. Clearly, the expected focusing and defocusing behavior is observed for the nominal wavelength, while caustics appear at 660 nm when the light is focused off-axis. Such chromatic aberrations can be efficiently predicted by a ray-optics description of the SP lens effect, as provided in ref (53) and reviewed for completeness in Section 1 of the Supporting Information. Notably, we observe a variation in intensity of the electron-induced near field right above the grating plane, which consistently shows a decay toward the larger periodicities at the top and bottom of the converging and diverging metalenses, respectively. We attribute this to a change in the electron-near-field excitation efficiency as the dimensions of the grating bars are altered (for more information, see Section 5 of the Supporting Information).

To experimentally study the emission of cylindrical SP radiation wavefronts, we fabricated 20 μm × 20 μm metalenses according to the design parameters provided in Figure 1. In addition, a conventional SP grating was fabricated as a reference sample with a constant 189 nm period. The structures were patterned into a 40 nm thin gold film using focused ion beam (FIB) milling (FEI Helios, Thermo Fisher Scientific Inc.), with the gold deposited by electron beam evaporation onto a flat 400 μm thick undoped silicon substrate with a 3 nm Cr adhesion layer (see Figure 2). We highlight that the properties of SP radiation are rather robust against defects,^63^ assuring that the desired emission properties can be achieved even in the presence of minor milling imperfections at the grain boundaries of the gold film.

**Figure 2:**
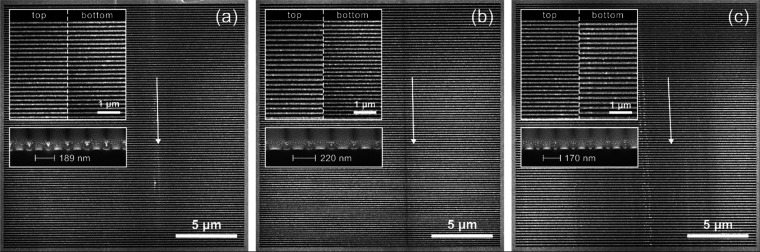
Fabricated Smith–Purcell metalens structures. (a) Reference grating with 189 nm spatial periodicity and a 50% fill factor. (b) Converging and (c) diverging metalenses of opposite chirps with a pitch ranging from 163 to 228 nm and a 50% fill factor, corresponding to a design focal length of ±17 μm at a nominal emission wavelength of 580 nm. The reference grating was chosen to yield dominant SP emission into a spectral range similar to that of its chirped counterparts. The top insets in each panel show the variation of the periodicity within the last few micrometers above and below the top and bottom of the structures, respectively. The bottom insets show FIB cross sections through the grating lines near the top, with a layer of platinum deposit used to increase the imaging contrast. White arrows indicate vertical traces of carbon contamination seen as dark lines that are deposited by the grazing electrons perpendicular to the grating lines. Chains of brighter spot-like deposits are also observed, presumably corresponding to larger carbon accumulations during the beam–sample alignment process.

The experimental far-field characterization of our metalenses and the reference grating was performed in a scanning electron microscope (FEI Quanta FEG 650, Thermo-Fisher Scientific Inc.) fitted with a commercial light collection setup for a spectral and angle-resolved cathodoluminescence analysis (SPARC, Delmic BV).^62^ As schematically depicted in Figure 3, light was collected from inside the microscope via an off-axis parabolic mirror, with the sample vertically mounted from below and parallel to the optical axis of the light collection optics. By careful adjustment of the sample tilt, the effective electron–sample interaction length *L*_eff_ was optimized to exploit the full length *L* = 20 μm of the grating structures and thus achieve SP emission within the maximum possible NA.

**Figure 3:**
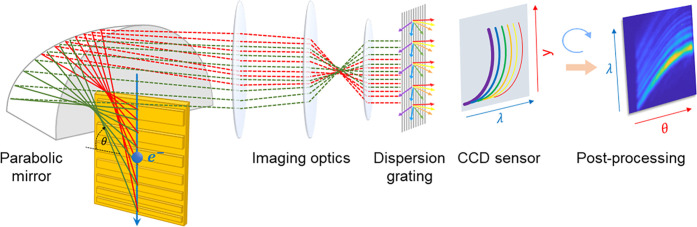
Hyperspectral angle-resolved light collection setup. The experimental setup is designed for simultaneous characterization of the far-field spectrum and angular emission profile of a sample upon electron beam excitation. A 3.3 nA, 30 keV electron beam passes through a 400 μm hole at the top of a parabolic mirror with a 2.5 mm focal length, carved into a solid aluminum block. The mirror is cut 500 μm above its focal plane, enabling light collection from a minimum zenithal emission angle of θ_min_ = 11° (with respect to the direction perpendicular to the electron beam). The grating structures are aligned parallel to the optical axis of the parabolic mirror, by piezoelectric actuators with sub-micrometer precision (controlling the position of the mirror) and a mechanical micrometer stage (controlling sample placement and tilt). The electron beam is aligned to graze the grating structures at a distance of a few nanometers distance, with the upper edge of the gratings positioned less than 10 μm below the mirror focus. Light emitted parallel to the sample plane (rays depicted as solid lines) is collected and collimated by the paraboloid (rays depicted as dashed lines). The collimated rays are then imaged onto a 100 μm wide slit (not shown) at the entrance of a spectrometer by a three-lens optical telescope, only transmitting light that is collected within a narrow azimuthal angular range around the mirror center. Subsequently, the light is dispersed by a diffraction grating and scattered onto a CCD sensor, where the horizontal and vertical positions onto the CCD sensor plane translate into a wavelength and emission angle (conversion between *y* and θ, as detailed in Section 7 in the Supporting Information). We note that, although the light collected in this configuration is emitted almost parallel to the grating surface, all relevant features of the metalens emission patterns are retained, as is also evident from the simulations shown in Figure 4.

Alignment of the sample with respect to the focal point of the parabolic mirror was performed by collecting incoherent cathodoluminescence from the uncoated top edge of the silicon substrate. The electron beam was then translated to graze the surface of the grating structures at a distance of a few nanometers. As further depicted in Figure 3, our light collection setup was operated in hyperspectral angle-resolved detection mode,^35^ simultaneously acquiring the far-field spectral response and the angular emission profile (with respect to the elevation angle θ = Θ – 90°) of our metalenses in a single measurement. Subsequently, the raw experimental data were corrected for the collection solid angle and spectral system response, as described in Section 8 of the Supporting Information. In accordance with the simulations discussed above, all measurements were performed at an excitation electron energy of 30 keV.

The left-hand panel in Figure 4a shows the measured dispersion of conventional SP radiation as emitted by the reference grating, in good agreement with the analytical expression given in eq 1 for Λ = 189 nm (red dashed line). Remarkably, the narrow width of the dispersion curve on the order of a few nanometers (fixed-angle cross section shown in the right-hand panel) indicates grazing of the electron beam along the entire length of the sample (*L*_eff_ ≈ 20 μm), leading to the coherent interference of light waves that are emitted by more than 100 grating periods.

**Figure 4:**
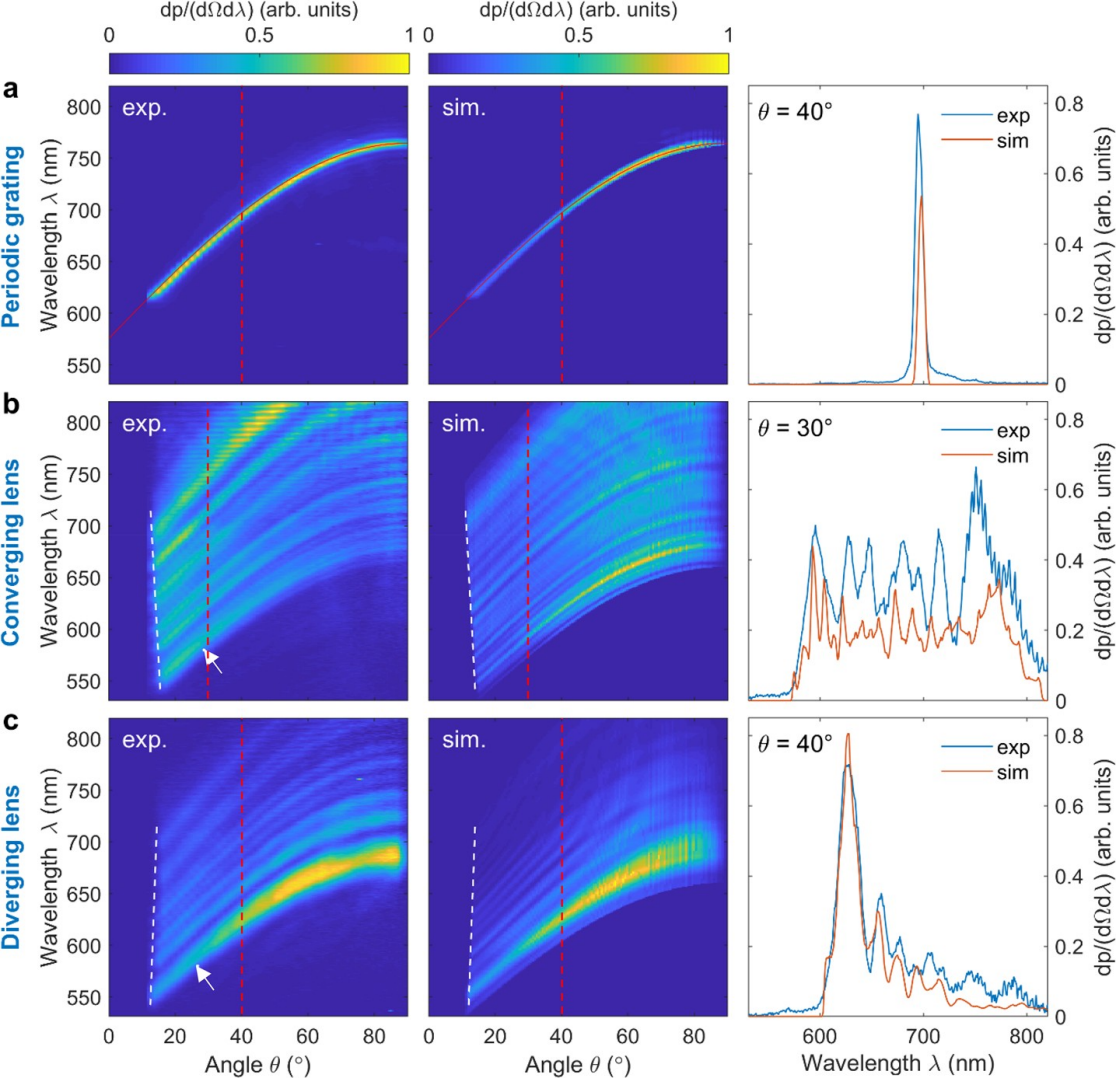
Far-field observation of SP focusing/defocusing (30 keV electron excitation). (a) Experimental (left) and simulated (middle) hyperspectral angle-resolved emission patterns of a conventional (periodic) SP grating (the red dashed line follows the SP dispersion according to eq 1 with Λ = 189 nm) and (right) fixed-angle cross section at θ = 40°, revealing a narrow spectral width on the order of a few nanometers for both the measurement (blue line) and simulation (red line). (b) Experimental (left) and simulated (middle) hyperspectral angle-resolved emission patterns of the converging Smith–Purcell metalens. Collection aberrations cause a negative slope at the left-hand edge of the spectrum (white dashed line). The arrow indicates the angular cutoff for emission in the nominal wavelength of 580 nm, resulting in an estimated NA of 0.48 ± 0.05, in good agreement with the design value of 0.5. The right panel is the fixed-angle cross section for θ = 30° showing characteristic spectral oscillations with similar relative amplitudes over almost identical bandwidths in both the simulations (red line) and measurements (blue line) (angle cross-section marked as a red dotted line, angle chosen to show best fit). (d) Experimental (left) and simulated (middle) hyperspectral angle-resolved emission patterns of the diverging Smith–Purcell metalens. In this case, collection aberrations cause an opposing positive slope at the left-hand edge of the spectrum (white dashed lines). The arrow indicates the angular cutoff at 580 nm, resulting in an estimated NA value of 0.45 ± 0.05. The right panel shows the fixed-angle cross section for θ = 40° extracted from simulations (red line) and measurement (blue line). Again, the experimental and numerical data appear in good agreement with respect to the position, width, relative amplitude, and envelope of prominent spectral features, with the best agreement occurring close to the nominal design wavelength λ_0_ of 580 nm. However, toward the upper spectral cutoff, the measurements reveal relative emissions into longer wavelengths slightly larger than those predicted by the simulations for both the converging and diverging metalenses.

In contrast, the radiation patterns of our converging and diverging metalenses presented in the left panels of Figure 4b,c are characterized by *broad-band* emission for any fixed angle θ, modulated by distinct oscillations. As opposed to the regular SP grating, the local momentum modulation of the grating near field along the electron trajectory gives rise to a multitude of closely spaced radiation bands with the typical SP dispersion curvature, each originating from a different subsection of the metalenses. The similar spectral bandwidths observed for both structures further attests to a sustained electron–near field interaction along the entire sample length.

To further interpret the above data, we resort to a hybrid simulation approach that combines the near-field distributions obtained by our full-wave numerical near-field simulations shown in Figure 1, with a geometrical ray-tracing analysis (Zemax OpticStudio) of the experimental light collection setup. The results of this procedure are presented in the middle panels of Figure 4. For the reference grating, a narrow dispersion curve is observed similarly to that in the experiments, featuring excellent overlap with the theoretical dispersion relation for Λ = 189 nm (red dashed line). Likewise, the simulation results for the converging and diverging metalenses match well with the oscillatory emission patterns observed experimentally, extending over similar spectral and angular ranges (see cross sections at selected angles shown in the right-hand panels of Figure 4b,c). In Section 2 of the Supporting Information, we additionally provide an analytical theory that accurately predicts the oscillatory behavior of the observed emission patterns. We show that this effect can be reduced to a phase-matching condition between the electron and the grating near field as briefly addressed above. The excellent agreement of the experimental data with both theory and simulations thus provides strong indirect evidence for the desired curved emission wavefronts and corresponding near-field effects of focusing and defocusing.

Moreover, our simulations reveal two key features that clearly distinguish the emission of the converging and diverging SP metalenses. First, the hyperspectral measurements show a slope in the λ–θ plane marked by the dashed white lines in Figure 4b,c. This measured feature is in full agreement with the hybrid simulation approach—showing an opposite trend for each type of lens and thus indicating sensitivity to the wavefront curvature. The opposite trends arise from a slight defocusing in the imaging system (such as an on-axis shift of the detector plane) that causes opposing aberrations for opposite incoming phase fronts.

Second, the observed emission patterns, and particularly the spectral power distribution, are highly sensitive to the grazing angle between the metalenses and the incident electron beam. We attribute this to the rapid evanescent decay of the grating near field upon interaction with the electron as the grazing distance is gradually increased. For positive grazing angles, this results in an enhanced emission into the longer and shorter wavelengths from the top sections of the converging and diverging metalenses, respectively. For negative grazing angles, an opposite trend may be expected; however, the interaction can be abruptly truncated upon collision of the electron with the sample surface, such that no light is emitted from grating sections farther down the electron trajectory. In experiment, a range of grazing angles can be attributed to both beam divergence and a minor tilt in the beam–sample alignment. By carefully comparing simulations and measurements, we find that the experimental features are best recovered by assuming a divergent electron beam comprised of multiple trajectories with grazing angles varying between −0.05 and +0.05° (for both lens types).

We note that the observed spectral variations in the emission probability discussed above are also consistent with a ray-optics description^53^ that combines the local SP dispersion of each finite subsection of the grating. In this picture, the coherent generation of different emission wavelengths is correlated with different positions along the grating, with the orientation of the rays being uniquely determined by the local SP dispersion. Thus, the correlation between emitted wavelength and position, as consistently observed in experiments, agrees well with the expected convergence or divergence of the emitted light. This discussion is further detailed in Section 3 of the Supporting Information, including reference measurements and analytical model predictions for electron incidence under grazing angles as well as complementary simulations for varying degrees of beam divergence.

For a quantitative analysis of the metalens emission patterns, we determine an effective NA by identifying the upper and lower cutoff emission angles at the nominal design wavelength of 580 nm (taken to be the midpoint between the angles corresponding to 10% and 90% of the maximum intensity, with the spacing between them taken as the uncertainty). The measured cutoff angles of the converging and diverging lenses yield NA values of 0.48 ± 0.05 and 0.45 ± 0.05, respectively, in good agreement with the design value of 0.5. In Section 5 of the Supporting Information we further provide an estimate of the metalens radiation efficiency.

To complement the conceptual findings of our experimental study, let us further illustrate the potential of metasurfaces as a tool to manipulate free-electron light emission and sculpture its spatial distribution by exploring combined control over the wavefront and polarization of SP radiation. For this purpose, we theoretically study a split-ring-resonator (SRR) metasurface with a chirped periodicity as defined by eq 2, rendering a cylindrical metalens with arbitrary tunable polarization.^54,64^ SRR meta-atoms are chosen due to their low-order symmetry as well as their bianisotropic nature,^65^ permitting coupling between electric and magnetic modes. Remarkably, the SRR meta-atoms thus allow for efficient focusing of light with a polarization component along the direction orthogonal to the electron trajectory (horizontal polarization in Figure 5a), in stark contrast to conventional SP radiation in which this polarization state is fully suppressed.

**Figure 5:**
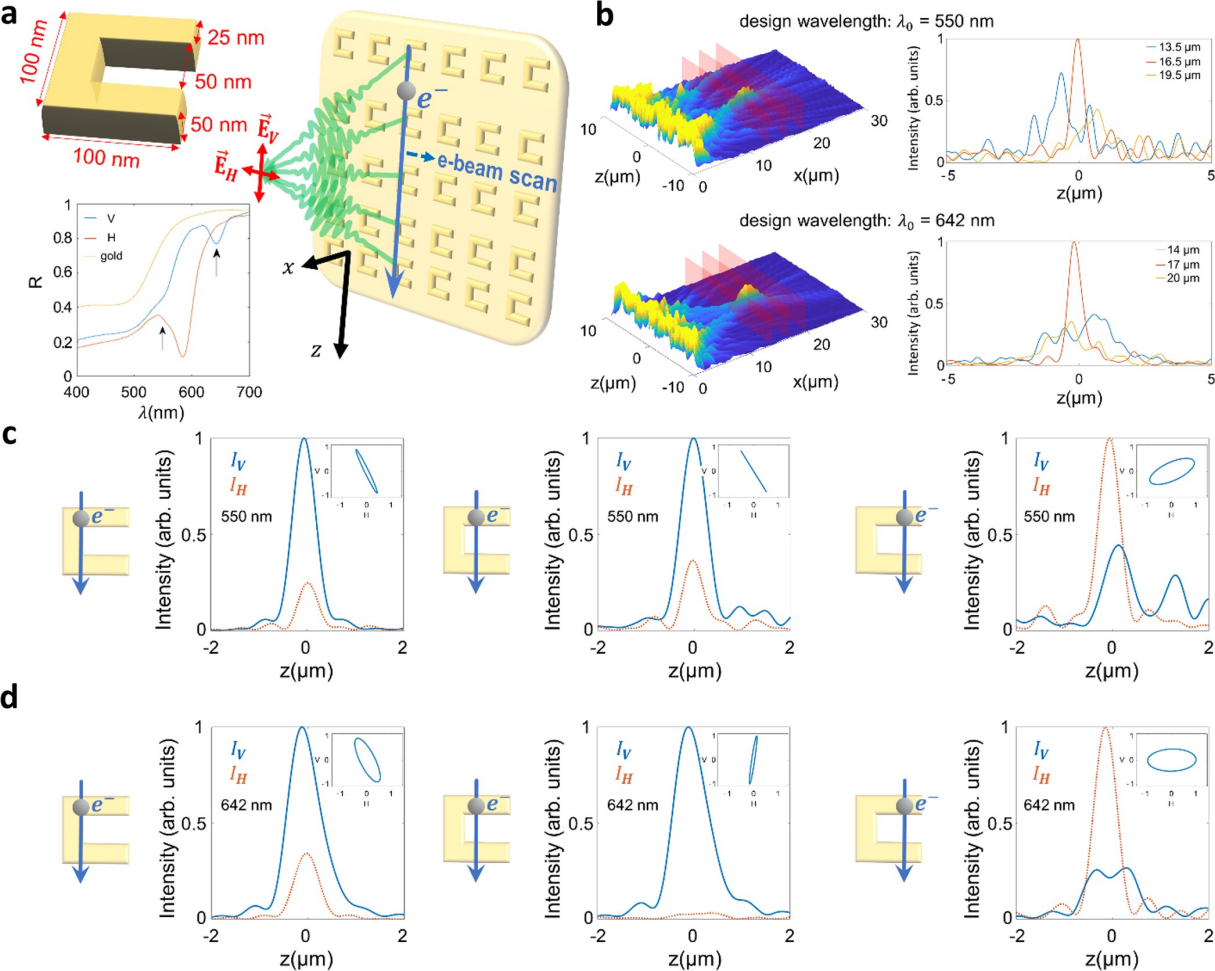
Proposal to use SRR metasurfaces for Smith–Purcell metalensing with highly controllable polarization. (a) Top right inset: schematic illustration of an SRR metalens, excited by a tightly collimated electron beam that is scanned along the horizontal direction relative to an array of SRR meta-atoms. Due to the bianisotropy of the SRRs, light is emitted into both vertical and horizontal polarizations. Top left inset: model geometry of an SRR meta-atom. Bottom left inset: periodic SRR metasurface reflection spectra upon excitation by a plane wave under normal incidence with a horizontal (red curve) or vertical polarization (blue curve). The reflection spectrum of a planar gold film is shown for reference (orange curve). Arrows mark two working points for metalenses with corresponding nominal design wavelengths of λ_0_ = 550 nm and λ_0_ = 642 nm. (b) Left: normalized intensity of the electron-induced optical field along the *x*–*z* plane for the two metalenses. Right: cross sections at approximately one Rayleigh range before and after and within the focal plane (marked in red on the left). (c, d) Focal intensity profiles of the vertical and horizontal polarization components for different electron impact parameters relative to the center of the SRR meta-atoms (−25, 0, and +25 nm). Data in (c) and (d) correspond to the metalenses with working points λ_0_ = 550 nm and λ_0_ = 642 nm, respectively. Insets: polarization ellipses of the light emitted into the focal spot.

The SRR geometry, depicted in Figure 5a, was optimized using numerical FDTD simulations of the focusing effect for the horizontal polarization at a nominal emission wavelength of λ_0_ = 550 nm and 30 keV incident electron energy. The bottom inset in Figure 5a shows reflection spectra of the SRR meta-atom for excitation by a plane wave under normal incidence that is polarized along the horizontal and vertical directions, respectively. For the horizontal polarization (red curve), the intended working point λ_0_ = 550 nm is situated to the left of a reflectivity dip at 584 nm, which we avoid to ensure efficient far-field scattering. For the vertical polarization (blue curve), however, a desirable dip in reflection occurs at λ_0_ = 642 nm, admitting another working point for a polarization-tunable metalens based on the same SRR meta-atom but with an adjusted periodicity. The left-hand panels of Figure 5b show the near-field intensity distributions obtained for the two different metalenses, clearly revealing the desired focusing effect at the respective nominal design wavelengths. For reference, the right-hand panels show 2D intensity cross sections as extracted from the focal plane, as well as at distances of approximately one Rayleigh length before and after.

Figure 5c,d shows a quantitative analysis of the polarization state of the emitted light within the focal plane (i.e., the intensity contributions by the vertical and horizontal polarizations and the corresponding polarization ellipse), as a function of the electron beam position upon grazing the SRR meta-atoms, for both lens designs. Notably, the polarization possesses a growing horizontal component for an electron beam trajectory closer to the gap edge of the SRRs, resulting from enhanced coupling to modes with a horizontal dipole moment (see Section 7 of the Supporting Information for a more thorough analysis of the coupling process and its effect on the emitted polarization).

In summary, we have fabricated and experimentally characterized a converging and a diverging free-electron-driven metalens based on the emission of Smith–Purcell radiation from an aperiodic metagrating. Our hyperspectral angle-resolved far-field measurements were found to be in good agreement with the results of numerical simulations as well as analytical model predictions, indirectly capturing the desired lens functionalities and revealing numerical apertures as high as 0.48 ± 0.05 and 0.45 ± 0.05, respectively. Our results provide the first experimental evidence that the wavefront of SP emission can be arbitrarily controlled by tailoring the shape of a metagrating, with a focusing effect as a specific example.

The concept of spatially modulated SP radiation demonstrated here could be utilized to produce efficient, focused light sources in the X-ray and extreme-ulltraviolet (EUV) regimes, possibly enhanced by material^66,67^ or structural resonances.^68,69^ Notably, the metalens design concept can be directly applied even in such extreme spectral regimes, by exploiting fabrication approaches based on layer deposition of nanometer or sub-nanometer thickness.^61^ Furthermore, we emphasize that the same concept may be transferred to the inverse Smith–Purcell effect,^70^ where the energy and shape of an electron beam could be altered by a laser wavefront impinging on an appropriate metastructure.

To put our work into perspective, we emphasize the vast number of degrees of freedom that can be attained by merging metasurface design principles with phase-matched free-electron radiation phenomena. As opposed to typical conventional far-field illumination schemes, free-electron-driven metasurfaces have the potential to simultaneously incorporate various functionalities and generate tunable radiation with virtually arbitrary spatial, spectral, temporal, and polarization properties. We emphasize that these prospects fundamentally arise from the highly localized nature of the electron–light–matter interaction with respect to both space and time (as opposed to a dipole or plane-wave excitation source). Structures such as the proposed two-dimensional SRR metasurface permit a transverse scanning of a tightly focused electron beam over its relative position with respect to the meta-atom and over different meta-atom arrays, with different shapes, resonances, and periodicities. The interaction with individual arrays of meta-atoms provides control over the angular, frequency, and even temporal response, thus significantly increasing the number of light source functionalities. Moreover, using widely collimated low-current electron beams or transversely wide multielectron bunches could permit the simultaneous excitation of different meta-atom arrays along the transverse dimension. In the first case, the metaatom arrays will emit incoherently,^71,72^ enabling amplitude modulation, while in the second case, excitations will interfere coherently, allowing combined transverse and longitudinal shaping of the emitted radiation.

## Supporting Information Available

Ray-optics description of Smith–Purcell metalenses, analytical treatment of Smith–Purcell near-field coupling, Smith–Purcell excitation at grazing angled incidence, description of numerical FDTD simulation, discussion on SP excitation efficiency, discussion on geometrical variations in the electron–near-field coupling efficiency, discussion on SRR meta-atom near-field excitations, and spectral and angular system response calibration (PDF)
