# Supplementary material for: Cylindrical Metalens for Generation and Focusing of Free-Electron Radiation
Source: Nano Lett. 2022 Jul 6;22(14):5641–50. doi: 10.1021/acs.nanolett.1c04556 (PMC9335868; doi:10.1021/acs.nanolett.1c04556)
Supplement: Supplementary file 1 — nl1c04556_si_001.pdf [file nl1c04556_si_001.pdf]

Supplemental Information:
Cylindrical metalens for generation and focusing of free-electron radiation

Aviv Karnieli[1†], Dolev Roitman[1†], Matthias Liebtrau[2†], Shai Tsesses[3†], Nika van Nielen[2], Ido Kaminer[3], Ady Arie[4*], and Albert Polman[2]

[1]Raymond and Beverly Sackler School of Physics and Astronomy, Tel Aviv University, Ramat Aviv 69978, Tel Aviv, Israel
[2]Center for Nanophotonics, NWO-Institute AMOLF, Science Park 104, 1098 XG Amsterdam, The Netherlands
[3]Andrew and Erna Viterbi Department of Electrical Engineering, Technion – Israel Institute of Technology, Haifa 32000, Israel
[4]School of Electrical Engineering, Fleischman Faculty of Engineering, Tel Aviv University, Tel Aviv 69978, Israel
[*]Corresponding author: ady@tauex.tau.ac.il
[†]equal contribution

SI1. Ray-optics description of Smith Purcell metalenses.

The design and operation of a Smith Purcell metalens can be described by a ray-optics approach as proposed in Ref. [1]. In this picture, the wave front of Smith Purcell radiation is shaped by introducing a relation between the angle Θ that a ray encloses with the direction of electron propagation, and the position z from which it is emitted along the electron trajectory. Assuming a generic phase profile $\phi(z)$ and a nominal emission wavelength λ_0, we can write this relation as [1]

$$\cos\Theta(z) = \frac{\lambda_0}{2\pi}\frac{d\phi(z)}{dz},$$

which upon insertion into the conventional SP relation $\lambda = \Lambda(\beta^{-1} - \cos\Theta)/m$ yields a spatially varying grating periodicity, with $\beta = v/c$ the normalized electron velocity and m the diffraction order. To model our metalenses, we introduce a cylindrical phase profile $\phi(z) = \mp 2\pi\sqrt{z^2+f^2}/\lambda_0$, with the negative and positive signs representing converging and diverging wave fronts, respectively. The corresponding periodicity profile then reads

$$\Lambda(z) = \frac{m\lambda_0}{\beta^{-1} \pm \frac{z}{\sqrt{z^2+f^2}}}, \tag{S1}$$

as given by Eq. (3) of the main text.

We can now apply the above ray-optics formalism to study chromatic aberrations of the metalenses at off-nominal wavelengths $\lambda \neq \lambda_0$. To this end, we insert Eq. (S1) back into the general SP relation with $m = 1$ and solve for the emission angle, yielding an expression of the form

$$\cos\Theta(z) = \mp\frac{\lambda}{\lambda_0}\frac{z}{\sqrt{z^2+f^2}} + \left(1 - \frac{\lambda}{\lambda_0}\right)\beta^{-1}.$$

For emission angles near the surface normal, we introduce the substitution $\theta = \Theta - \pi/2$, and apply a small-angle approximation to find

$$\theta(z) \cong \mp\frac{\lambda z}{\lambda_0 f} + \left(1 - \frac{\lambda}{\lambda_0}\right)\beta^{-1} = \mp\frac{z}{\tilde{f}(\lambda)} + \Delta\theta(\lambda)$$

with $\tilde{f}(\lambda) = \frac{\lambda_0}{\lambda}f$ and $\Delta\theta(\lambda) = \left(1 - \frac{\lambda}{\lambda_0}\right)\beta^{-1}$. It follows that the emission of the metalenses is subject to both longitudinal and lateral chromatic aberrations as represented by the parameters $\tilde{f}(\lambda)$ and $\Delta\theta(\lambda)$,

respectively. These aberrations show opposing trends for red and blue shifts $\Delta\lambda$ relative to λ_0, as also illustrated in Fig. S1a for $\Delta\lambda = \pm\ 30$ nm and $\lambda_0 = 580$ nm. In addition, the model reveals a distorted caustic nature of the focusing effect at off-nominal wavelengths, in good agreement with the rigorous full-wave numerical near-field simulations shown in Fig. 1 of the main text. Notably, since $\Delta\theta(\lambda)$ scales inversely proportional to the electron velocity, the lateral chromatic aberrations can be compensated for a given wavelength by adjusting the electron energy. We demonstrate this effect in Fig. S1b by the example of a metalens with a nominal design wavelength of 580 nm at 5 keV excitation electron energy, showing a caustic off-axis focus for emission at 590 nm wavelength. We find that by increasing the electron energy to 30 keV, the desired focal spot is recovered, and aberrations are largely eliminated. However, we note that due to the wave nature of the emitted light, focusing is limited by diffraction, and that an Airy-beam-like pattern is introduced to the caustic focusing effect at off-nominal wavelengths.

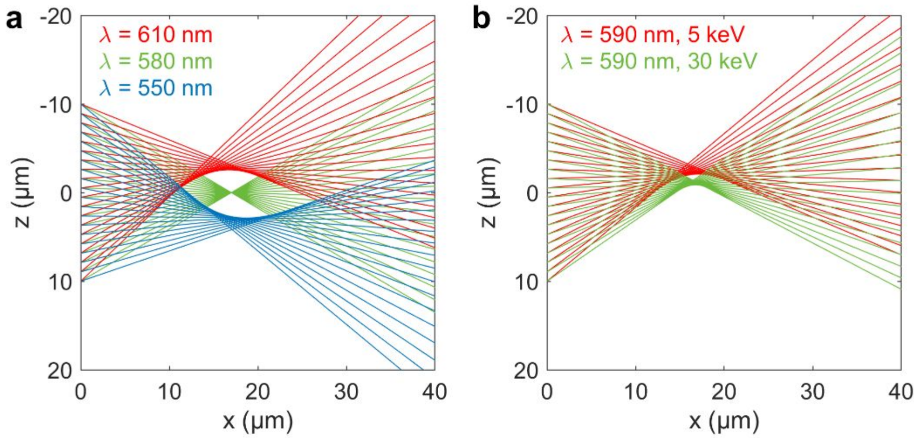

Fig. S1: **Ray-optics model of the Smith Purcell metalens. a** Rays emitted by a converging lens with a nominal design wavelength $\lambda_0 = 580$ nm, for different wavelengths $\lambda = 550$ nm, 580 nm, and 610 nm. For the off-nominal wavelengths, we observe both lateral and longitudinal aberrations, combined with a caustic distortion of the focus. **b** Chromatic aberration correction by tuning the electron energy. A converging metalens with nominal design wavelength $\lambda_0 = 580$ nm at 5 keV incident electron energy shows slight chromatic aberrations at $\lambda = 590$ nm. The aberrations are corrected by increasing the electron energy to 30 keV.

SI2. Analytical treatment of Smith Purcell near-field coupling.

In the following, we derive an analytical framework that allows us to correlate the far-field characteristics of our Smith Purcell metalenses with the underlying electron-near-field coupling mechanism. To this end, we consider first a generic photonic system that interacts with a point-like free electron of kinetic energy E_0. The probability for the electron to excite the system at frequency ω and thus suffer a corresponding energy loss $\hbar\omega \ll E_0$ can be written as [2]

$$p_{\mathrm{loss}} = \left| \frac{e}{\hbar\omega} \int_{-\infty}^{\infty} \mathrm{d}z\, \boldsymbol{\mathcal{E}}(\mathbf{r}_{\perp,0}, z) \cdot \hat{\mathbf{z}}\, e^{-i\frac{\omega}{v}z} \right|^2, \tag{S2}$$

where e is the electron charge, v is the electron velocity, $\hat{\mathbf{z}}$ is the unit vector in the direction of electron propagation, $\boldsymbol{\mathcal{E}}(\mathbf{r}_\perp, z)$ is the electron-induced electric field amplitude and $\mathbf{r}_{\perp,0} = (x_0, y_0)$ denotes the transverse electron coordinates. The coupling integral governing Eq. (S2) corresponds to the Fourier transformation of the electric field as projected onto the electron trajectory at an electron-velocity-dependent spatial frequency $q = \omega/v$. Physically, this imposes a phase-matching condition which can be only fulfilled if light is slowed down by either material dispersion or boundary conditions, i.e. in the near-field of a nanostructure.

In our Smith Purcell experiments, the measured radiation patterns are determined by the far-field envelope $\boldsymbol{\mathcal{E}}_\infty(\mathbf{r},\omega)$ of the electron-induced electric field $\boldsymbol{\mathcal{E}}(\mathbf{r},\omega)$ in Eq. (S2). Similar to the electron energy loss probability p_{loss}, the patterns thus ultimately depend on the electron-light coupling efficiency as shown below. To this end, we consider grating structures that are patterned along the z direction, in line with the convention used in the main text. For an observer that is located at a large distance r away from the source, we can write the measured differential photon emission probability as [3]

$$\frac{d^2 p_{\mathrm{rad}}}{d\Omega d\omega} = \frac{r^2 \varepsilon_0 c}{\pi \hbar \omega} |\boldsymbol{\mathcal{E}}_\infty(r\hat{\mathbf{n}},\omega)|^2,$$

where $\hat{\mathbf{n}}$ is the direction of observation, $d\Omega$ denotes an infinitesimal small collection solid angle element, and ε_0 is the vacuum permittivity. Under the simplifying assumption of a lossless medium, we may choose to expand the electric field in terms of normal modes $\mathbf{u}_{\mathbf{k}}$ [3] which are uniquely defined by the boundary-value problem imposed by the grating geometry. This permits an ansatz according to the following considerations: The near field of a regular SP grating has to inherit the grating periodicity Λ, giving rise to Fourier waves with excess momentum $m\hbar k_g \hat{\mathbf{z}}$, where m is the harmonic order of the reciprocal grating constant $k_g = 2\pi/\Lambda$. For the chirped metalenses, the periodicity slowly varies along z, thus the near field acquires a local phase that can be approximated by an integral of the form $m\int_0^z \mathrm{d}z' \frac{2\pi}{\Lambda(z')}$. For orders $|m| > 0$, the excess momentum provided by the different grating geometries ensures phase-matching between the electron and the light wave while causing an evanescent decay in the direction perpendicular to the grating plane, here taken to be the x direction. Hence, the normal modes of the periodic reference grating and the chirped metalenses admit expressions of the form

$$\mathbf{u}_{\mathbf{k}}(\mathbf{r}) = \begin{cases} \sum_m \mathbf{A}_m(\mathbf{r}_\perp,\mathbf{k}) e^{i\left(\sqrt{k^2 - k_y^2 - \left(k_z + \frac{2\pi}{\Lambda}m\right)^2}\, x + k_y y + \left(k_z + \frac{2\pi}{\Lambda}m\right) z\right)}, & \text{periodic grating,} \\ \sum_m \mathbf{A}_m(\mathbf{r}_\perp,\mathbf{k}) e^{i\left(\sqrt{k^2 - k_y^2 - \left(k_z + \frac{2\pi}{\Lambda(z)}m\right)^2}\, x + k_y y + k_z z + m\int_0^z \mathrm{d}z' \frac{2\pi}{\Lambda(z')}\right)}, & \text{metalens.} \end{cases}$$

Importantly, we stress that the mode index $\mathbf{k} = k\hat{\mathbf{n}}$ refers to the plane-wave ($m = 0$) component of the modal wave vector, i.e. the component which is eventually scattered into the far-field. Resorting to a classical Green's function formalism, in the Appendix we derive Eq. (S3) in terms of these normal modes as

$$\frac{d^2 p_{\mathrm{rad}}}{d\Omega d\lambda} = \frac{\alpha}{\lambda^3} |\mathbf{A}_0(k\hat{\mathbf{n}})|^2 \left| \int \mathrm{d}z\, \mathbf{u}_{k\hat{\mathbf{n}}}(\mathbf{r}_{\perp,0},z) \cdot \hat{\mathbf{z}}\, e^{-i\frac{\omega}{v}z} \right|^2, \qquad \text{(S3)}$$

where $|\mathbf{A}_0(k\hat{\mathbf{n}})|^2$ (the Fourier coefficient of the plane-wave component of the mode) captures the material- and geometry-dependent angular radiation pattern of mode $\mathbf{k}$, $\alpha = e^2/4\pi\epsilon_0\hbar c$ is the fine structure constant, and $k = 2\pi/\lambda = \omega/c$. Notably, under the given assumptions, the above expression suggests that only a single mode $\mathbf{u}_{k\hat{\mathbf{n}}}(\mathbf{r})$ contributes to the far-field amplitude of light emitted into a given direction of observation $\hat{\mathbf{n}}$ and wavelength λ. Moreover, we find that up to a different normalization, the dependence of Eq. (S3) on the modal near-field is indeed equivalent to the form of the electron energy loss probability given by Eq. (S2), in good agreement with a rigorous quantum mechanical treatment of the problem for small dipolar emitters [4]. Substituting the above ansatz with $k_z = k\cos\Theta$, and limiting ourselves to a contribution by the $m = 1$ Fourier component (corresponding to first-order Smith-Purcell emission), we eventually find

$$\frac{d^2p_{\text{rad}}}{d\Omega d\lambda} = \frac{\alpha}{\lambda^3}|\mathbf{A}_0(k\hat{\mathbf{n}})|^2|\mathbf{A}_1(\mathbf{r}_{\perp,0},k\hat{\mathbf{n}})\cdot\hat{\mathbf{z}}|^2 \begin{cases} \left|\int_{-\infty}^{\infty} \mathrm{d}z e^{-\kappa x_0(z)} e^{ik\left(\cos\Theta - \beta^{-1} + \frac{\lambda}{\Lambda}\right)z}\right|^2, \text{ grating} \\ \left|\int_{-\infty}^{\infty} \mathrm{d}z e^{-\kappa x_0(z)} e^{ik\left(z\cos\Theta - z\beta^{-1} + \int dz\frac{\lambda}{\Lambda(z)}\right)}\right|^2, \text{metalens} \end{cases},\text{(S4)}$$

where $\kappa \approx \frac{2\pi}{\lambda}\sqrt{\beta^{-2}-1}$ defines the evanescent decay of the near-field amplitude away from the grating plane. We note that in the above expressions the electron is permitted an incident grazing angle $\alpha_t \neq 0$ such that $x_0(z) = x_0 + \tan\alpha_t(z - z_0)$.

In general, the Fourier coefficients defining the terms $|\mathbf{A}_1(\mathbf{r}_T,k\hat{\mathbf{n}})\cdot\hat{\mathbf{z}}|^2$ and $|\mathbf{A}_0(k\hat{\mathbf{n}})|^2$ introduce a highly non-trivial dependence of the metalens emission patterns on both the observation direction $\hat{\mathbf{n}}$ and emission wavelength λ. Thus, for a quantitative comparison between theory and experiment, we resort to a numerical solution of Maxwell's equations in the main text, taking into account the details of the metalens geometry and material dispersion. However, fortunately, the relevant features associated with the electron near-field coupling mechanism are fully recovered by the remaining integral terms. To analyze this most clearly, we will continue assuming a vanishing grazing angle $\alpha_t \approx 0$, while a complementary discussion of angled grazing incidence is provided in section SI3.

From the upper entry of Eq. (S4), we immediately recover the conventional first-order SP dispersion relation through a delta function $\frac{d^2p_{\text{rad}}}{d\Omega d\lambda} \propto \delta\left(\lambda - \Lambda(\beta^{-1} - \cos\theta)\right)$, strictly reflecting the electron-light phase-matching condition addressed above. In the measurements, spectral broadening is introduced by a finite interaction range L, as also reproduced by a truncation of the integral in Eq. (S4). This is illustrated in Fig. S2a, using the structural parameters of our reference grating. The lower entry of Eq. (S4) is further specified by substituting $\Lambda(z)$ from Eq. (S1), permitting us to write the local phase accumulation of the first order grating mode ($m = 1$) as

$$\int_0^z \mathrm{d}z' \frac{2\pi}{\Lambda(z')} = \frac{2\pi}{\lambda_0}\int\left(\frac{c}{v} \pm \frac{z}{\sqrt{z^2+f^2}}\right)dz = \frac{2\pi}{\lambda_0}\left(\frac{c}{v}z \pm \sqrt{z^2+f^2}\right). \qquad \text{(S5)}$$

Thus, we finally obtain an expression of the form

$$\frac{d^2p_{\text{rad}}}{d\Omega d\lambda} \propto \left|\int_{-L/2}^{L/2} \mathrm{d}z e^{i\left(\frac{2\pi}{\lambda}\cos\Theta + \frac{2\pi}{\lambda_0}\left(1 - \frac{\lambda_0}{\lambda}\right)\beta^{-1}\right)z} e^{\pm i\frac{2\pi}{\lambda_0}\sqrt{z^2+f^2} - \kappa(\lambda)x_0(z)}\right|^2, \qquad \text{(S6)}$$

where the integral was adapted to the finite physical extent of the metalenses. Calculated probability patterns for the converging and diverging metalenses per the design parameters used in the main text are shown in Fig. S2b and c, respectively. The theory accurately reproduces the occurrence of multiple curved emission bands over a wide spectral bandwidth, in good agreement with the experimental data. However, as opposed to the measurements, the calculated radiation patterns are identical for the two types of metalenses. This is because the opposing curvature of the emission wavefronts cannot be distinguished based on their far field intensity, but requires additional phase information. Thus, under ideal conditions as assumed in theory, the metalens emission patterns only differ in the near-field (see simulations in Fig. 1 in the main text). Yet, as detailed in the main text, we stress that in practice the wavefront curvatures can be told apart owing to defocusing aberrations of the experimental light collection setup. Furthermore, in the following section we show that a non-zero grazing angle leaves an opposite signature on the far-field emission of the two lens types.

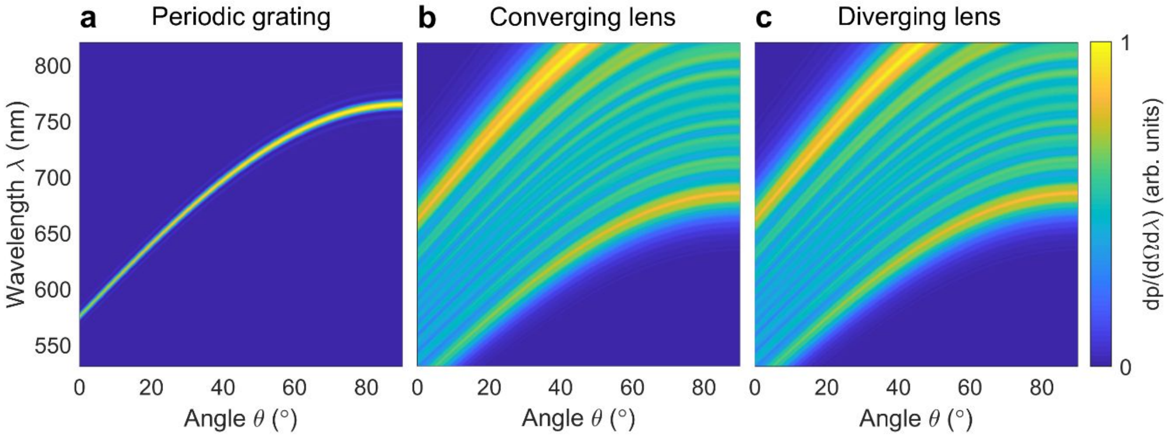

Fig. S2: **Analytical model.** Calculated radiation patterns of **a** the periodic reference grating, **b** the converging metalens, and **c** the diverging metalens considering only the contribution of electron-near-field phase-matching. Calculations are according to the design parameters used in the main text, assuming 30-keV electron excitation at a constant grazing distance of $x_0 = 20$ nm. For the metalenses, the theory accurately predicts a distribution over multiple curved emission bands, in good agreement with the experimental observations. However, under the given assumptions, there are no far-field features distinguishing the two lens types (these are discussed in section SI3).

SI3. Smith Purcell excitation at grazing angled incidence.

In our experiments, the radiation patterns of the converging and diverging metalenses are clearly distinguished by their spectral intensity distributions. As demonstrated below, the main factor responsible for this difference can be identified as a non-vanishing grazing angle between the incident electron beam and the sample plane, as well as a finite beam divergence. To analyze this effect, in the following, we distinguish *positive* (i) and *negative* (ii) grazing angles for which the electrons propagate away from and towards the sample plane, respectively.

Using our analytical model theory derived in section SI2, in Fig. S3 we show a comparison between the metalens radiation patterns for perfect grazing incidence with those predicted for small *positive* (i) grazing angles of $0.05°$ and $0.1°$. In this case, electrons are passing closer by the top of the metalenses than by the bottom, causing a rapid exponential decay of the electron-near-field coupling according to Eq. (S6). As a result, the converging and diverging metalens radiation patterns show opposing spectral skews towards the longer and shorter emission wavelengths, respectively. Notably, this effect is in good agreement with the linear relation between the SP emission wavelength and the position along the grating, namely, the local grating period that is prevalent in the interaction. This suggests that different radiation bands can be associated with different sections of the metalenses, and that their intensity varies due to the aforementioned change in the near-field coupling with position. However, we note that the analytic theory does not recover spectral features associated with material dispersion or the metalens geometry, requiring an analysis in terms of numerical simulations as provided in the main text.

To illustrate the effect of *negative* (ii) grazing angles, Fig. S4 shows experimental data for $\alpha_t \approx$ -1˚ to -2˚. In this case, the interaction is terminated upon collision of the electrons with the sample plane at just a few microns from the top of the gratings. As a result, the converging and diverging metalens radiation patterns are strongly skewed towards the long and short emission wavelengths, respectively, in good agreement with the theory. In contrast, the emission of the regular SP grating maintains overlap with the expected SP dispersion relation, given that the grating period is constant. However, we note that a shorter interaction length results in both spectral broadening and a reduced radiation efficiency. Moreover, we observe that the emission probability is gradually damped towards decreasing emission wavelengths. For grazing distances on the order of a few tens of nanometers, this could be related to the

exponential decay term in Eqs. (S4), predicting stronger coupling to near-field components with a larger wavelength and thus a smaller evanescent decay constant κ.

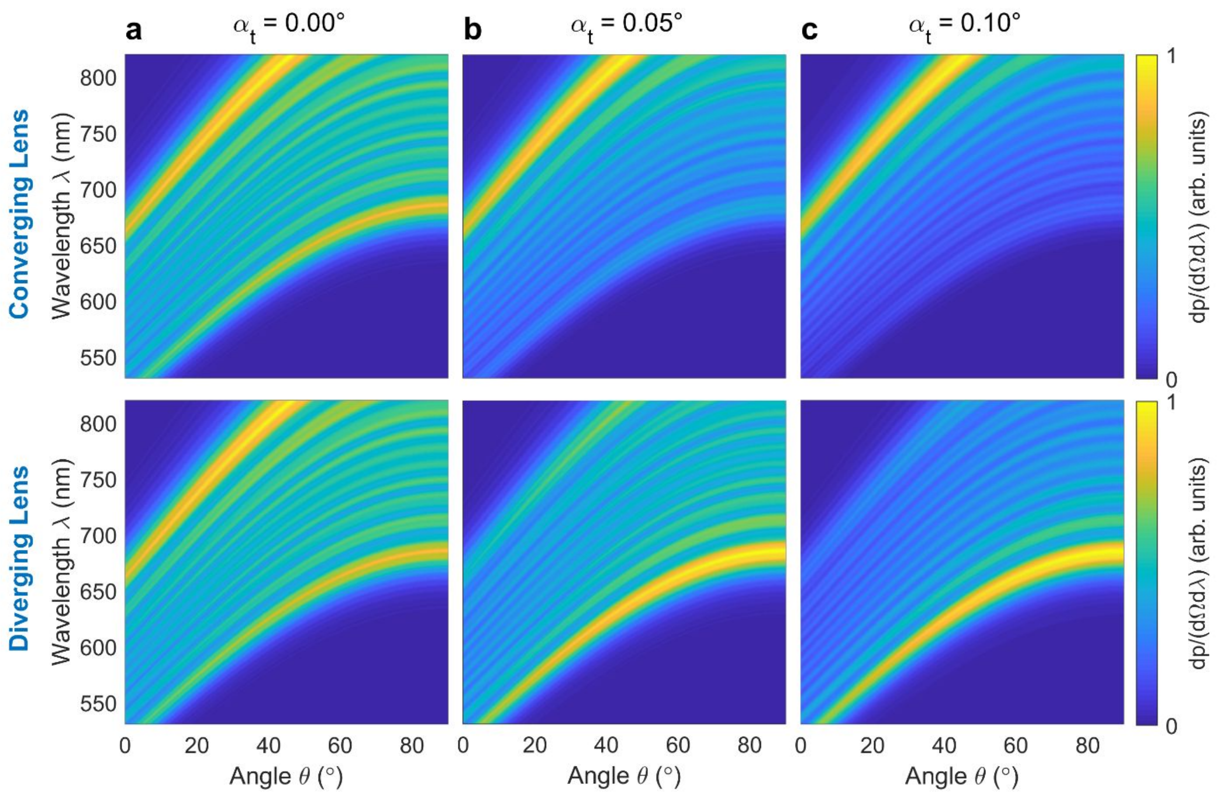

Fig. S3 **Metalens radiation patterns for electron incidence under small *positive* grazing angles.** Analytical model calculations assuming 30 keV electron excitation at a minimum grazing distance of 20 nm. For increasing grazing angle, we consistently observe a skew of the converging and diverging metalens radiation patterns towards the long and short emission wavelengths, respectively. This can be related to an exponential dependence of the SP excitation efficiency with the grazing distance, as is evident from Eq. (S6).

Finally, we consider a combination of *both positive* and *negative* grazing angles, allowing us to emulate the effect of a divergent electron beam. To also account for the combined effects of material dispersion and geometrical features, we performed numerical simulations for a range of electron trajectories that span grazing angles α_t between $-0.05°$ and $0.05°$, and $-0.1°$ and $0.1°$. The resulting radiation patterns are plotted in comparison to the experimental data in Fig. S5. For reference, the results obtained for perfect grazing incidence are also shown. Notably, we observe clear spectral trends that are consistent with the intuition gained by our analytical model theory. The best agreement between experiment and simulations is found assuming a range of angles $-0.05° \leq \alpha_t \leq 0.05°$, as stated in the main text. This corresponds to a beam divergence angle smaller than 2 mrad, constituting a reasonable value in scanning electron microscopy [5]. Yet, we conclude that the exact form of the acquired metalens radiation patterns is very sensitive to a combination of parameters including the grazing distance, grazing angle, beam divergence but also the exact sample geometry and material dispersion.

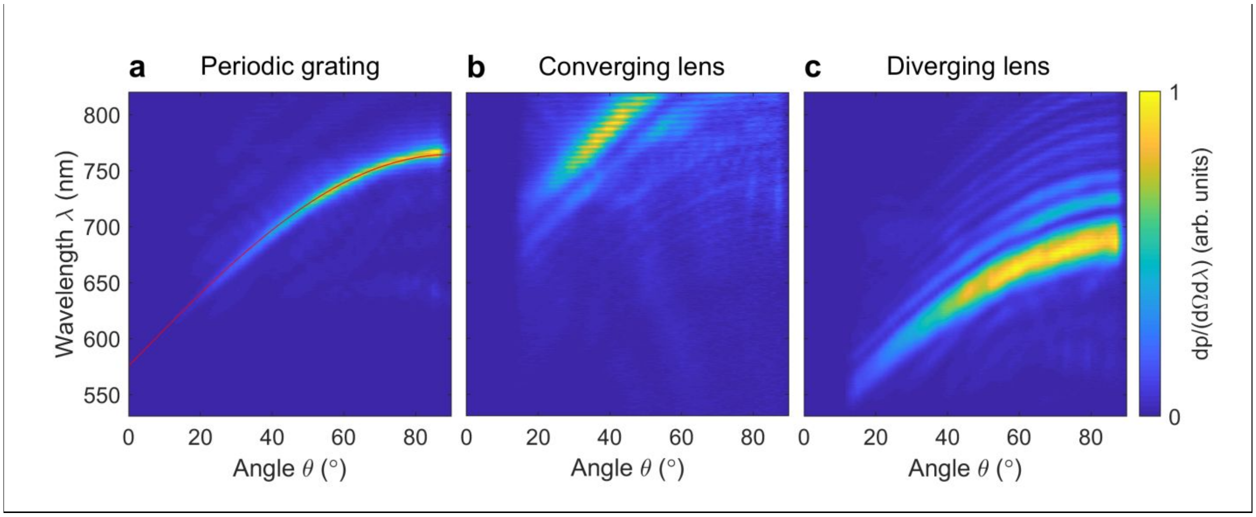

Fig. S4: **Experimental SP radiation patterns for truncated electron-beam-sample interaction at negative grazing angles.** A 30-keV electron beam excites **a** the reference grating, **b** the converging metalens, and **c** the diverging metalens at a small *negative* grazing angle α_t of -1˚ to -2˚. In this configuration, the beam axis intersects with the sample plane after a few microns distance from the top of the structures, abruptly truncating the interaction. For reference, the red line in **a** represents the theoretical SP dispersion for a grating with a 189 nm period.

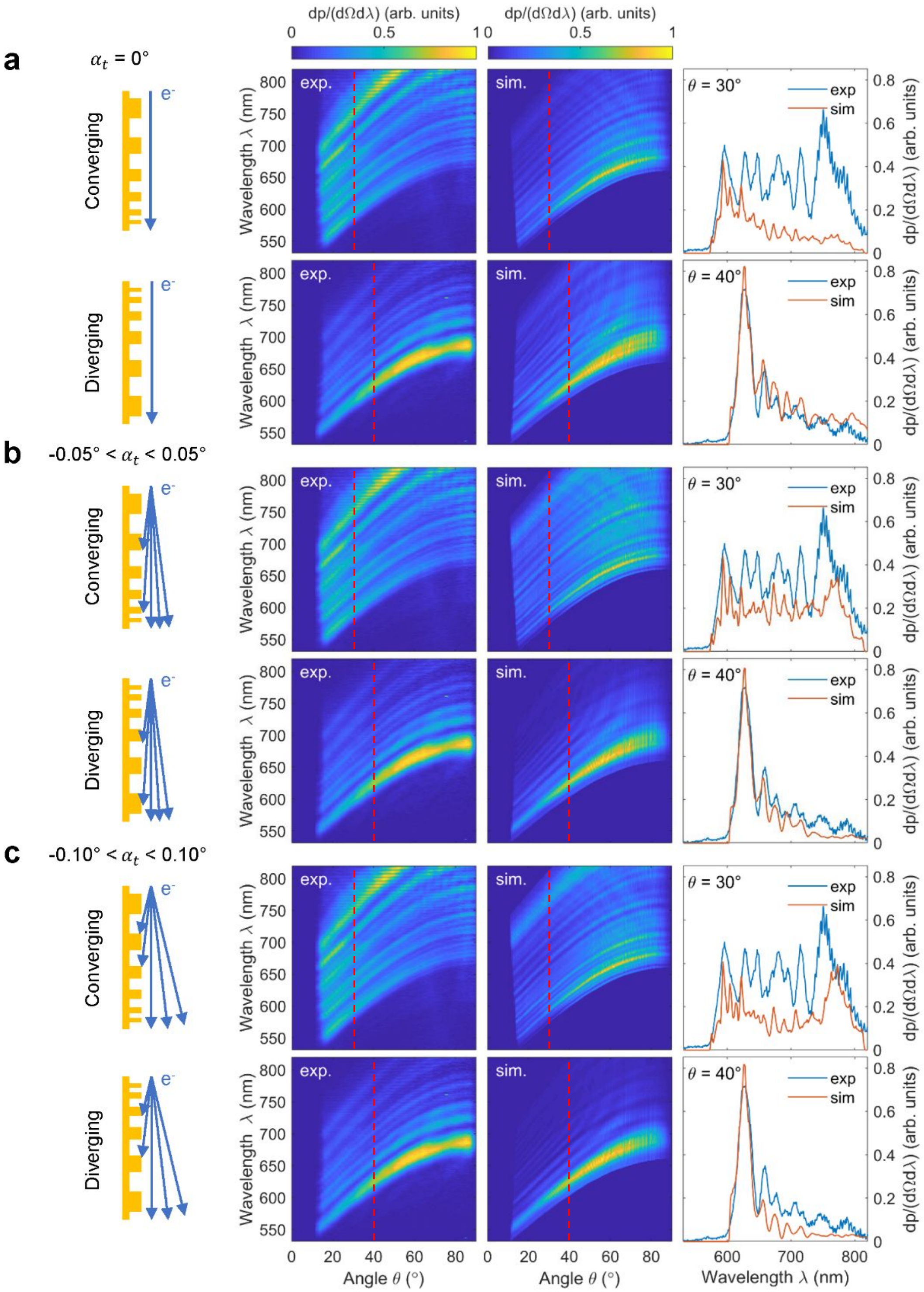

Fig. S5: **Numerical hybrid simulations of metalens radiation patterns for divergent electron beam excitation.** Each simulation incorporates 5 electron trajectories within the angular range specified in **a**, **b**, and **c**. The best agreement between simulations and the experimental data is obtained for a beam divergence of $-0.05° \le \alpha_t \le 0.05°$ (as also seen from the emission spectra for selected angles shown on the right). As in the experiments, numerical data are obtained for 30 keV incident electron energy, with the grazing distance assumed to be 5 nm.

SI4. Numerical FDTD simulations.

To numerically simulate free-electron radiation over a broad spectral range, we resort to a commercial three-dimensional full-wave finite-difference in the time domain (FDTD) solver (Lumerical Inc., FDTD solutions). The electron beam excitation was emulated by an array of dipoles with a phase-lag determined by the electron velocity v. For a point-like electron propagating along the z direction with transverse coordinates $\mathbf{r}_{\perp,0} = (x_0, y_0)$ we can write the associated beam current as

$$\mathbf{j}(\mathbf{r},\omega) = \hat{\mathbf{z}} e \delta(\mathbf{r}_\perp - \mathbf{r}_{\perp,0}) e^{i\frac{\omega}{v}z},$$

while a point-like dipole is represented by a dipole-moment of the form

$$\mathbf{d}(\mathbf{r},\omega) = e^{i\frac{\omega}{v}z_0} \mathbf{d}(\omega) \delta(\mathbf{r}_\perp - \mathbf{r}_{\perp,0}) \delta(z - z_0).$$

Thus, by choosing $\mathbf{d}(\omega) = d(\omega)\hat{\mathbf{z}}$ we obtain a source current of the form

$$\mathbf{j}(\mathbf{r},\omega) = d(\omega) \hat{\mathbf{z}} \delta(\mathbf{r}_\perp - \mathbf{r}_{\perp,0}) \sum_n e^{i\frac{\omega}{v}z_n} \delta(z - z_n)$$

with z_n spaced by a distance much smaller than the wavelength (5 nm). The electron-induced nearfield was recorded using a frequency domain monitor placed 1 µm away from the electron source, either parallel or perpendicular to the grating plane (depending on the orientation of the assumed collection). To reveal the focusing and defocusing effects shown in Fig. 1 of the main text, the recorded vectorial fields were numerically propagated in free space to a range of distances from 1 µm to 40 µm. The far-field data used for the Zemax ray-tracing analysis in Fig. 4 of the main text were obtained by a software integrated near-to-far-field transformation algorithm.

SI5. SP excitation efficiency.

As detailed in ref. [6], the excitation efficiency for visible and near-infrared SP radiation by electrons of 5-30 keV in an SEM can approach the order of 10^{-3}-10^{-4}. In our numerical simulations, the electron excitation is modeled by a chain of dipole sources with a phase-lag determined by the electron velocity, inducing an evanescent field (see previous section). Thus, we can relate the optical power radiated into the far-field to the combined power of all dipole sources with a total of 4 pW. Fig. S6 shows the emitted spectral power density, both for the periodic reference grating and for the converging metalens, using the design parameters defined in the main text. We assume perfect grazing electron incidence at 30 keV kinetic energy with a total interaction range of 20 µm (close to the experimental conditions). By integrating over the corresponding spectra, we extract radiation efficiencies of $2.3 \times 10^{-3}$and 2.4×10^{-3} for the periodic grating and the converging metalens, respectively, in good agreement with the theoretical expectations.

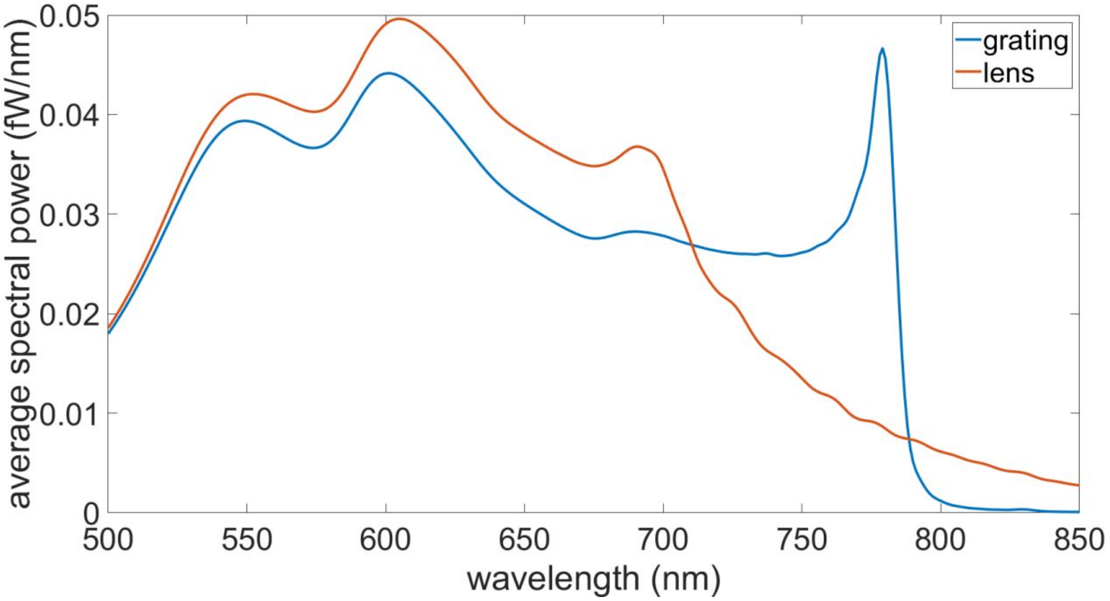

Fig. S6: **Numerically simulated power density spectra of the Smith-Purcell grating and the converging metalens.** In order to obtain the total SP excitation efficiency, the spectral power is integrated over all emission wavelengths and divided by the total dipole chain input power of 4 pW, yielding estimated efficiencies of $2.3 \times 10^{-3}$and 2.4×10^{-3}, respectively.

SI6. Geometrical variations in the electron-near-field coupling efficiency.

Close inspection of the near-field simulations shown in Fig. 1 of the main text reveals a variance in intensity along the surface of the metalenses. For a fixed emission wavelength, we can relate this effect to variations in the electron-near-field coupling efficiency as a function of the grating period, i.e. the exact spatial near-field distribution of the individual grating bars. To support this interpretation, Fig. S7 shows the electron-induced near-field intensity upon excitation of different gratings with periodicities in the range of 150 nm to 250 nm. Simulations are performed for a fixed emission wavelength of $\lambda = 580$ nm, assuming 30 keV incident electron energy at a grazing distance of 5 nm. The data qualitatively capture the intensity variations observed near the surface of the metalenses, showing a similar decay with increasing period. For completeness, we note that the coupling efficiency also scales with the emission wavelength as determined by the evanescent decay constant κ of the near-field away from the grating plane in Eq. (S6).

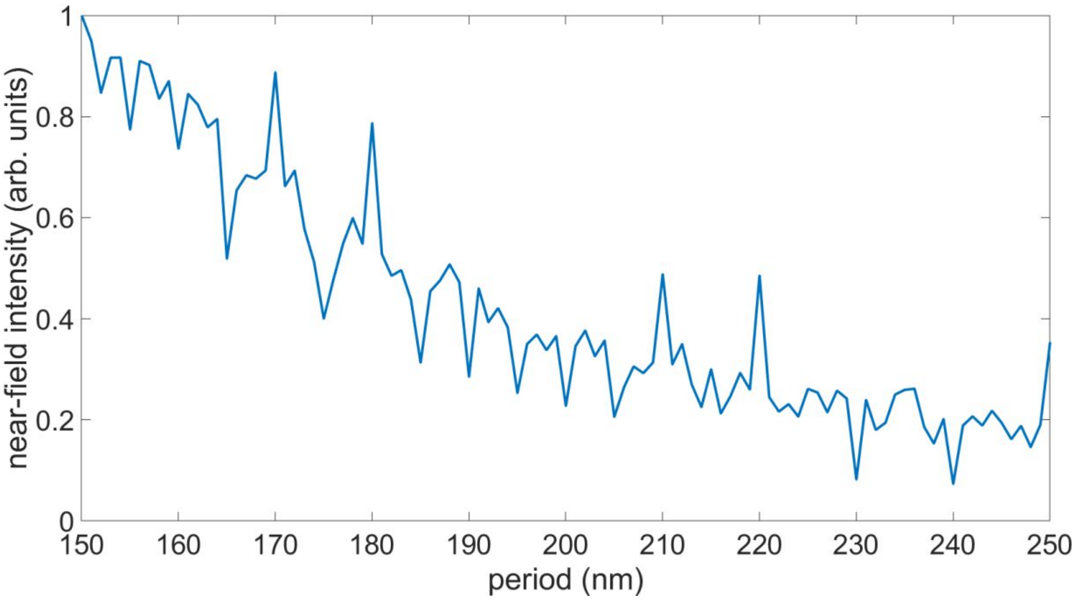

Fig. S7: **Electron-induced near-field intensity of an SP grating as a function of the grating period.** Simulations are performed assuming excitation at 30 keV incident electron energy, a grazing distance of 5 nm, and $\lambda = 580$ nm emission wavelength. Clearly, we see a global decrease in near-field intensity with the grating period, in good agreement with the intensity variations observed a few nanometers from the surface of the metalenses in Fig. 1 of the main text.

SI7. SRR meta-atom near-field simulations.

For the subsequent discussion, we consider a decomposition of the SRR meta atoms into two parts – a dimer of rod-like antennas connected by a wire which forms another rod-like antenna (a similar structure was rigorously explored in ref. [7]). For an electron passing near the gapless edge, the induced near field is expected to be governed by the modes of the connecting rod. Thus, the SRR would primarily emit vertically (V) polarized radiation, similar to the emission from an extended grating ruling. On the other hand, for an electron passing near the gap, we expect dominant coupling to the dimer modes, increasing the component of H polarization of the emitted radiation. This is corroborated by the polarization ellipse in Fig. 5 of the main text, as well as the total electron-induced near-field distributions shown in Fig. S8a at 550 nm excitation wavelength for the electron passing near the connecting wire and the gap, respectively. At the second chosen working point of 642 nm shown in the main text, there is a dip in the reflectivity for the V polarization (see the reflection analysis of Fig. 5 in the main text). Thus, in general, more energy is transferred to the near-field of the V-polarization mode (which does not necessarily indicate subsequent coupling to radiation). This effect seems to extend the SRR mode beyond the rod excitation, when the electron impinges at -25 nm relative to the SRR center (left). The dimer excitation continues to be dominant when the electron passes at +25 nm (right).

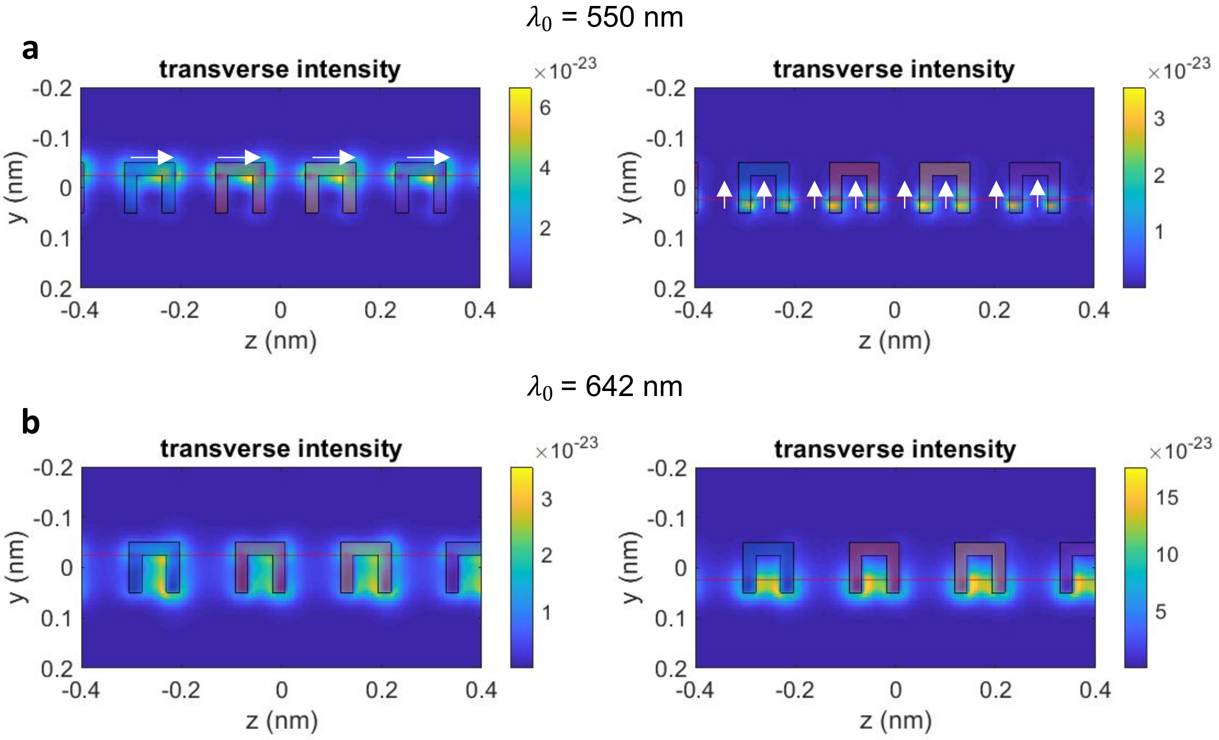

Fig. S8: **SRR near field excited by a passing electron at the two design wavelengths of $\lambda_0 = 550$ nm and $\lambda_0 = 642$ nm.** **a** for the $\lambda_0 = 550$ nm working point: the electron passes near the rod (left) or near the dimer (right) and excites dipole either along (left) or perpendicular to (right) its trajectory. **b** for the $\lambda_0 = 642$ nm: a near-resonant frequency (according to the reflection spectra of Fig. 5 in the main text). Here, more energy is coupled between the rod and dimer near field modes, even when the electron passes near the rod (left), but the far-field polarization is still dominantly vertical (along the electron trajectory). Right: near field distribution when the electron passes near the dimer, again exciting mostly horizontal far-field polarization.

SI8. Spectral and angular system response calibration.

The hyperspectral angle-resolved emission patterns shown in this work were acquired using a Czerny-Turner spectrograph equipped with a 500 nm blaze dispersion grating (300 lines/mm) and a back-illuminated charge-coupled device (CCD) sensor array (Andor Newton DU940P-BU). The system was aligned such that the spectral and angular information were projected onto the horizontal and vertical sensor axes, respectively. A vertical slit aperture with a horizontal width of 100 µm was introduced to filter light emission from a narrow azimuthal range around the optical axis of the parabolic light-collection mirror. Samples were exposed to an electron beam current of approximately 3.3 nA for durations of 300 s for the metalenses and 250 s for the reference grating.

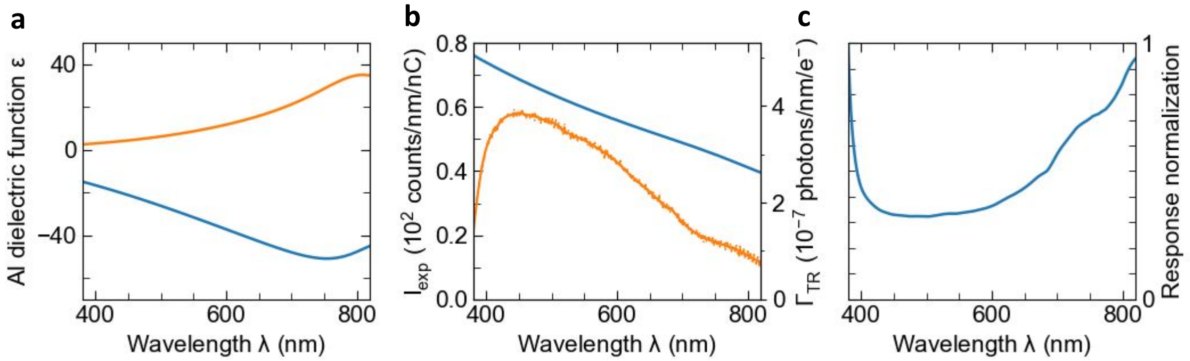

Fig. S9: **Spectral response calibration of the light collection setup. a** Real (blue) and imaginary part (orange) of the dielectric function of a mono-crystalline Al sample emitting transition radiation (TR)**. b** Integrated TR spectrum measured in hyperspectral angle-resolved light detection mode upon 30-keV electron beam excitation. A smoothening function (solid orange line) was applied to the experimental data I_{exp} (orange dotted curve) in order to avoid noise artefacts upon normalization to the calculated TR emission probability Γ_{TR} (solid blue line). **c** Inverted spectral response function of the light collection setup applied to normalize the measured intensity distributions.

The spectral system response function of the light collection setup was determined according to a similar procedure as described in Ref. [8]. In brief, transition radiation (TR) excited by a 30-keV electron beam was collected from the flat surface of a mono-crystalline aluminum sample and acquired in hyperspectral angle-resolved detection mode. The resulting emission pattern was integrated over all angles and normalized to the analytical TR expression given in Ref. [9]. For the calculations, optical constants representing the employed aluminum sample were derived from spectroscopic ellipsometry measurements (see Fig S9a). We stress that an angle-resolved spectral response calibration was omitted since the emission of TR is strongly suppressed in the direction of the surface normal [9]. However, considering a dominant contribution by the spectrograph (dispersion grating, CCD sensor), angular features were assumed to have little effect on the overall spectral system response.

Following Refs. [10-12], the vertical pixel arrays of the CCD sensor were calibrated for the zenithal emission angle θ by mapping the one-dimensional projection of the light collection mirror onto its parabolic contour in the plane spanned by the electron beam and the optical axis of the imaging system. For the n-th pixel in an array, the corresponding emission angle θ_n was derived from an expression of the form

$$\theta_n = \frac{\pi}{2} + \arctan\left(az_n - \frac{1}{4az_n}\right),$$

where a = 0.1 mm^{-1} is the parabola coefficient and z_n denotes the vertical coordinate of a mirror surface element above the focal plane. The latter was calculated as $z_n = z_0 + n(z_N - z_0)/(N+1)$, where $z_0 =$

0.5 mm and z_N = 11.5 mm correspond to the lower and upper mirror cutoffs, respectively, and $N+1$ is the total number of enclosed pixels. In addition, the hole for electron transmission provides a useful reference for upwards emission at an angle $\theta = 0$. Eventually, the calibrated data were normalized to the collection solid angle using an expression of the form

$$\mathrm{d}\Omega_\mathrm{n} \propto \frac{2az_n^2 - x_n}{\left(x_n^2 + z_n^2\right)^{\frac{3}{2}}}$$

with $x_n = az_n^2 - (4a)^{-1}$.

We note that the above procedure is optimized for light emission by a point source that is precisely aligned with the mirror focal plane. In our Smith Purcell experiments, however, light is emitted by elongated sources that extend more than 20 µm below the focus. Reassuringly, a ray-tracing analysis has revealed little distortion of the angular system response by the precise vertical sample placement. Nevertheless, we found significant chromatic aberrations that are correlated with the curvature of the emission wavefronts. For reference, this effect is also reproduced in Fig. S10, showing the emission patterns of our model structures as projected onto the CCD sensor plane, prior to the transformation of the vertical axis into angles. At the bottom, the patterns are truncated by the lower cutoff of the light-collection mirror, with opposing slopes distinguishing the two metalenses.

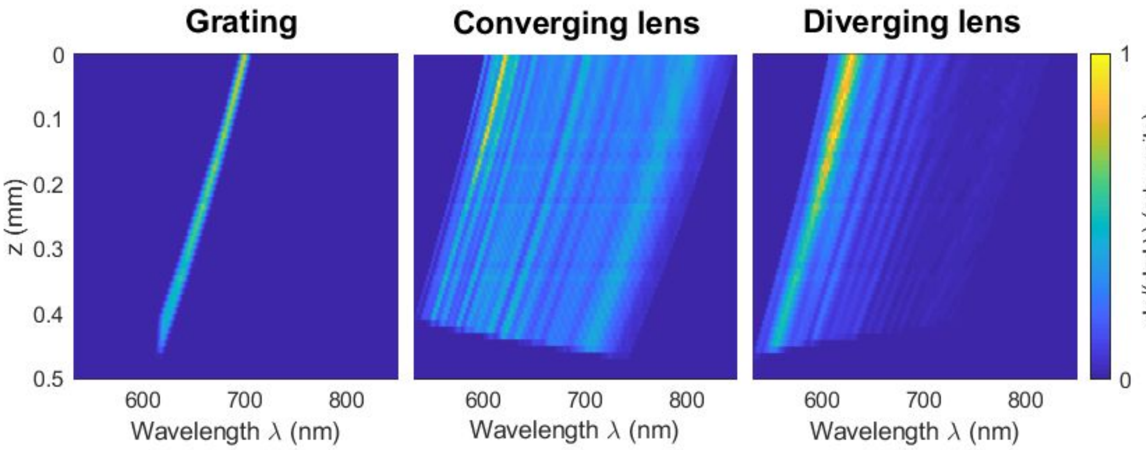

Fig. S10: **Raw ray-tracing projection of the simulated metalens and periodic grating radiation patterns onto a planar screen.** At the bottom, the metalens radiation patterns are truncated along a line with opposing slope, revealing wave-front-related chromatic aberrations in the projection of the lower cutoff of the parabolic light collection mirror.

Due to minor system imperfections, we note that the experimental data feature additional chromatic aberrations that manifest as a common slope in the projection of the hole for electron transmission. Thus, the measured patterns were calibrated by linear adaptation of the reference angles θ_0 and θ_N for each spectral data slice λ_m, with a small uncertainty of 1 to 2 pixels. However, to maintain and unambiguously recover wave-front related aberrations, we stress that the exact same calibration parameters were used for both the converging and diverging metalenses. In addition, the calibration of the reference SP grating was verified against the theoretical SP dispersion relation. The error introduced by the calibration uncertainty can be estimated as

$$\Delta\theta_n = \frac{a + \frac{1}{4az_n^2}}{1 + \left(az_0 - \frac{1}{4az_n}\right)^2}\Delta z = \frac{2az_n + \cot\theta_n \Delta z}{1 + \cot^2\theta_n \quad z_n},$$

where Δz is given by twice the size of the CCD sensor pixels of 13.5 µm for two-fold binning. Considering the lower cutoff angle $\theta_0 \approx 11°$, we obtain $\Delta\theta_0 \cong \tan\theta_0(\Delta z/z_0) \lesssim 1°$.

<u>Appendix:</u> Analytical derivation of electron-induced far-field emission probabilities

In the following, we apply an analytical Green's function formalism to derive the far-field emission probabilities of our Smith Purcell structures under the assumptions posed in section SI2. First, we note that the emitted far field amplitude $\boldsymbol{\mathcal{E}}_\infty(r\hat{\mathbf{n}},\omega)$ can be obtained from the angular spectrum representation of the electric field [3] on a reference plane $\mathbf{s}$, with $\hat{\mathbf{s}}$ denoting the surface normal, observed at a direction $\hat{\mathbf{n}}$:

$$\boldsymbol{\mathcal{E}}_\infty(r\hat{\mathbf{n}},\omega) = -2\pi i\frac{\omega}{c}\hat{\mathbf{n}}\cdot\hat{\mathbf{s}}\frac{e^{i\frac{\omega}{c}r}}{r}\int d^2\mathbf{s}\, e^{i\frac{\omega}{c}\hat{\mathbf{n}}\cdot\mathbf{s}}\boldsymbol{\mathcal{E}}(\mathbf{s},\omega)$$

By taking the reference plane $\mathbf{s}$ to be the transverse plane spanned by $\hat{\boldsymbol{\theta}}$ and $\hat{\boldsymbol{\phi}}$ (with $\hat{\mathbf{s}} = \hat{\mathbf{n}}$) we find

$$\boldsymbol{\mathcal{E}}_\infty(r\hat{\mathbf{n}},\omega) = -2\pi i\frac{\omega e^{i\frac{\omega}{c}r}}{c\ \ r}\int\int du dv \boldsymbol{\mathcal{E}}(u\hat{\boldsymbol{\theta}} + v\hat{\boldsymbol{\phi}},\omega),\ \text{(A1)}$$

Further, we can relate the far field to the source current $\mathbf{j}(\mathbf{r},\omega) = \hat{\mathbf{z}}e\delta(\mathbf{r}_\perp - \mathbf{r}_{\perp,0})e^{i\frac{\omega}{v}z}$ induced by a free electron for propagation along the z direction via [3]

$$\boldsymbol{\mathcal{E}}_\infty(r\hat{\mathbf{n}},\omega) = i\omega\mu_0\int d^3\mathbf{r}'\mathbf{G}_\infty(r\hat{\mathbf{n}},\mathbf{r}',\omega)\mathbf{j}(\mathbf{r}',\omega), \qquad \text{(A2)}$$

where $\mathbf{G}_\infty(r\hat{\mathbf{n}},\mathbf{r}',\omega)$ is the far-field dyadic Green's function. Assuming emission into a homogeneous medium with dielectric permittivity $\varepsilon_r(\mathbf{r},\omega)$, it proves useful to introduce normal modes $\mathbf{u_k}(\mathbf{r})$ that satisfy the relations $\nabla\times\nabla\times\mathbf{u_k}(\mathbf{r}) - \varepsilon_r(\mathbf{r},\omega)\frac{\omega^2}{c^2}\mathbf{u_k}(\mathbf{r}) = 0$ and $\int d^3\mathbf{r}\left[\frac{1}{2\omega}\frac{d(\omega^2\varepsilon_r(\mathbf{r},\omega))}{d\omega}\right]_{\omega=\omega_\mathbf{k}}\mathbf{u_k}(\mathbf{r})\mathbf{u_{k'}}(\mathbf{r}) = \delta_{\mathbf{kk'}}$ [3].

Thus $\mathbf{G}_\infty(r\hat{\mathbf{n}},\mathbf{r}',\omega)$ can be evaluated in terms of a mode expansion [3]

$$\mathbf{G}_\infty(r\hat{\mathbf{n}},\mathbf{r}',\omega) = \sum_\mathbf{k} c^2\frac{\mathbf{u}_{\mathbf{k},\infty}(r\hat{\mathbf{n}})\mathbf{u}_\mathbf{k}^*(\mathbf{r}')}{\omega_\mathbf{k}^2 - \omega^2}, \qquad \text{(A3)}$$

where $\mathbf{u}_{\mathbf{k},\infty}(r\hat{\mathbf{n}})$ denotes the far-field amplitude of mode $\mathbf{u_k}(\mathbf{r})$ with the summation running over the wave vector $\mathbf{k}$ of all allowed momentum states. In free space, these states are given by plane waves that approximately follow a continuous dispersion relation $\omega_\mathbf{k} = ck$, transforming the sum in Eq. (A3) into an integral. Choosing an ansatz for the grating modes $\mathbf{u_k}(\mathbf{r})$ as introduced in section SI2, insertion into Eq. (A1) yields the normalized far field amplitude $\mathbf{u}_{\mathbf{k},\infty}(r\hat{\mathbf{n}})$ in the explicit form

$$\mathbf{u}_{\mathbf{k},\infty}(r\hat{\mathbf{n}}) = -\frac{2\pi i e^{ikr}}{k\ \ r}\mathbf{A}_0(k\hat{\mathbf{n}})\delta(\hat{\mathbf{k}} - \hat{\mathbf{n}}).$$

We can now derive the far-field dyadic Green's function as

$\mathbf{G}_\infty(r\hat{\mathbf{n}},\mathbf{r}',\omega)$

$$= \int \frac{d^3k}{(2\pi)^3} c^2 \frac{\mathbf{u}_{\mathbf{k},\infty}(r\hat{\mathbf{n}})\mathbf{u}^*_{\mathbf{k}}(\mathbf{r}')}{\omega^2_{\mathbf{k}} - \omega^2} = -i\frac{1}{\omega}\frac{c^2}{8\pi^2}$$

$$\int dk d\Omega k \frac{e^{ikr}}{r} \mathbf{A}_0(k\hat{\mathbf{n}}) \delta(\hat{\mathbf{k}} - \hat{\mathbf{n}}) \mathbf{u}^*_{\mathbf{k}}(\mathbf{r}') \left(\frac{1}{\omega_{\mathbf{k}} - \omega - i\delta} - \frac{1}{\omega_{\mathbf{k}} + \omega + i\delta} \right)$$

$$= -i\frac{1}{\omega}\frac{c}{8\pi^2} \int dk k \frac{e^{ikr}}{r} \mathbf{A}_0(k\hat{\mathbf{n}}) \mathbf{u}^*_{k\hat{\mathbf{n}}}(\mathbf{r}') \left(\frac{1}{k - \frac{\omega}{c} - i\delta} - \frac{1}{k + \frac{\omega}{c} + i\delta} \right) = \frac{e^{i\frac{\omega}{c}r}}{4\pi r} \mathbf{A}_0\left(\frac{\omega}{c}\hat{\mathbf{n}}\right) \mathbf{u}^*_{\frac{\omega}{c}\hat{\mathbf{n}}}(\mathbf{r}')$$

where in the last step we exploited Cauchy's integral identity assuming a vanishing loss term $\delta \to 0$. By insertion into Eq. (A2), we obtain the electron-induced far-field in the form

$$\boldsymbol{\mathcal{E}}_\infty(r\hat{\mathbf{n}},\omega) = ie\omega\mu_0 \frac{e^{i\frac{\omega}{c}r}}{4\pi r} \mathbf{A}_0\left(\frac{\omega}{c}\hat{\mathbf{n}}\right) \int dz' e^{i\frac{\omega}{v}z'} \mathbf{u}^*_{\frac{\omega}{c}\hat{\mathbf{n}}}(\mathbf{r}_{\perp,0},z') \cdot \hat{\mathbf{z}}.$$

The corresponding power distribution per unit frequency and unit solid angle as collected by a detector can be written as :

$$\frac{d^2P}{d\Omega d\omega} = \frac{\alpha\hbar\ \omega^2}{4\pi^2 T c^2} \left| \mathbf{A}_0\left(\frac{\omega}{c}\hat{\mathbf{n}}\right) \right|^2 \left| \int dz' e^{i\frac{\omega}{v}z'} \mathbf{u}^*_{\frac{\omega}{c}\hat{\mathbf{n}}}(\mathbf{r}_{\perp,0},z') \cdot \hat{\mathbf{z}} \right|^2$$

where $\alpha = e^2/4\pi\epsilon_0\hbar c$ is the fine structure constant and $T = L/v$ is the interaction time. Finally, we obtain the desired emission probabilities with respect to the emission wavelength from

$$\frac{d^2p}{d\Omega d\lambda} = \frac{d\omega\ T}{d\lambda\ \hbar\omega} \frac{d^2P}{d\Omega d\omega} = \alpha |\mathbf{A}_0(k\hat{\mathbf{n}})|^2 \frac{1}{\lambda^3} \left| \int dz e^{-i\frac{\omega}{v}z} \mathbf{u}_{k\hat{\mathbf{n}}}(\mathbf{r}_{\perp,0},z) \cdot \hat{\mathbf{z}} \right|^2$$

where $k = 2\pi/\lambda$ and $\omega = 2\pi c/\lambda$.

References

1. Remez, R. *et al.* Spectral and spatial shaping of Smith-Purcell radiation. *Phys. Rev. A* **96**, 061801 (2017).
2. Di Giulio, V., Kociak, M. & García de Abajo, F. J. Probing quantum optical excitations with fast electrons. *Optica* **6**, 1524 (2019).
3. Novotny, L. & Hecht, B. Principles of Nano-Optics. Cambridge University Press, 2006.
4. Asenjo-Garcia, A. & García de Abajo, F. J. Plasmon electron energy-gain spectroscopy. *New J. Phys.* **15**, 103021 (2013).
5. Reimer, L. Scanning Electron Microscopy: Physics of Image Formation and Microanalysis. Second Edition, Springer, 1998.
6. Yang, Y. et al, Maximal spontaneous photon emission and energy loss from free electrons, *Nat. Phys.* **14** 894 (2018)
7. Zhang, S. *et al.* Plasmon-induced transparency in metamaterials. *Phys. Rev. Lett.* **101**, 047401 (2008).
8. Brenny, B. M. J., Coenen, T. & Polman, A. Quantifying coherent and incoherent cathodoluminescence in semiconductors and metals. *J. Appl. Phys.* **115**, 244307 (2014).
9. García de Abajo, F. J. Optical excitations in electron microscopy. *Rev. Mod. Phys.* **82**, 209-275 (2010).
10. S. Mignuzzi *et al.* Energy-momentum cathodoluminescence spectroscopy of dielectric nanostructures. *ACS Photon.* **5**, 1381 (2018).

11. Coenen, T. & Polman, A. Energy-momentum cathodoluminescence imaging of anistropic directionality in elliptical aluminum plasmonic bullseye antennas. *ACS Photon.* **6**, 573 (2019).
12. Schilder, N. J., Agrawal, H., Garnett, E. C. & Polman, A. Phase-resolved surface plasmon scattering probed by Cathodoluminescence Holography. *ACS Photon.* **7**, 1476 (2020).
